# Dramatic Acceleration of the Hopf Cyclization on Gold(111): From Enediynes to Peri-Fused Diindenochrysene Graphene Nanoribbons

**DOI:** 10.1021/jacs.3c10144

**Published:** 2024-01-16

**Authors:** Chenxiao Zhao, Dayanni D. Bhagwandin, Wangwei Xu, Pascal Ruffieux, Saeed I. Khan, Carlo A. Pignedoli, Roman Fasel, Yves Rubin

**Affiliations:** † Nanotech@surfaces Laboratory, Empa−Swiss Federal Laboratories for Materials Science and Technology, 8600 Dübendorf, Switzerland; ‡ Department of Chemistry and Biochemistry, University of California, Los Angeles, 607 Charles Young Dr. East, Los Angeles, California 90095-1567, United States; § Department of Chemistry, Biochemistry and Pharmaceutical Sciences, University of Bern, 3012 Bern, Switzerland

## Abstract

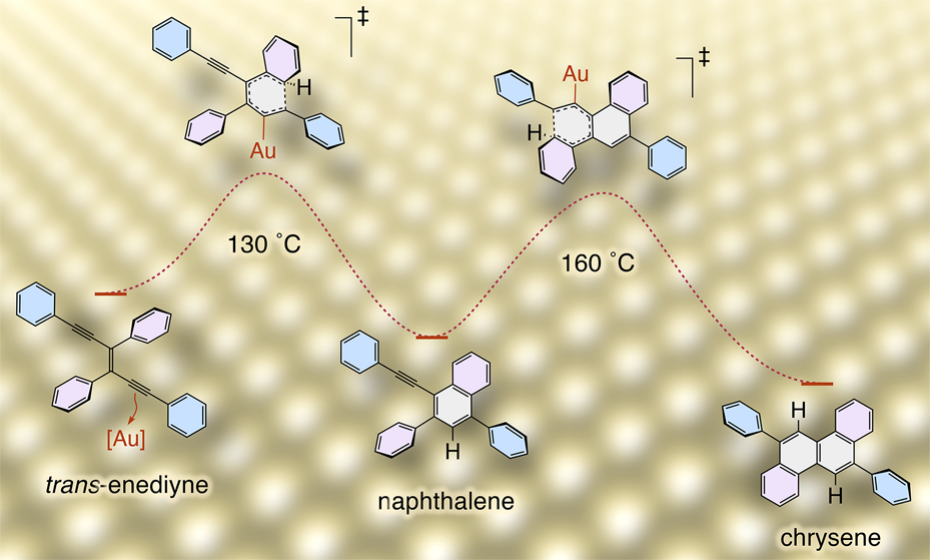

Hopf et al. reported the high-temperature 6π-electrocyclization of *cis*-hexa-1,3-diene-5-yne to benzene in 1969. Subsequent studies using this cyclization have been limited by its very high reaction barrier. Here, we show that the reaction barrier for two model systems, (*E*)-1,3,4,6-tetraphenyl-3-hexene-1,5-diyne (**1a**) and (*E*)-3,4-bis(4-iodophenyl)-1,6-diphenyl-3-hexene-1,5-diyne **(1b)**, is decreased by nearly half on a Au(111) surface. We have used scanning tunneling microscopy (STM) and noncontact atomic force microscopy (nc-AFM) to monitor the Hopf cyclization of enediynes **1a,b** on Au(111). Enediyne **1a** undergoes two sequential, quantitative Hopf cyclizations, first to naphthalene derivative **2**, and finally to chrysene **3**. Density functional theory (DFT) calculations reveal that a gold atom from the Au(111) surface is involved in all steps of this reaction and that it is crucial to lowering the reaction barrier. Our findings have important implications for the synthesis of novel graphene nanoribbons. Ullmann-like coupling of enediyne **1b** at 20 °C on Au(111), followed by a series of Hopf cyclizations and aromatization reactions at higher temperatures, produces nanoribbons **12** and **13**. These results show for the first time that graphene nanoribbons can be synthesized on a Au(111) surface using the Hopf cyclization mechanism.

## Introduction

**Figure 1:**
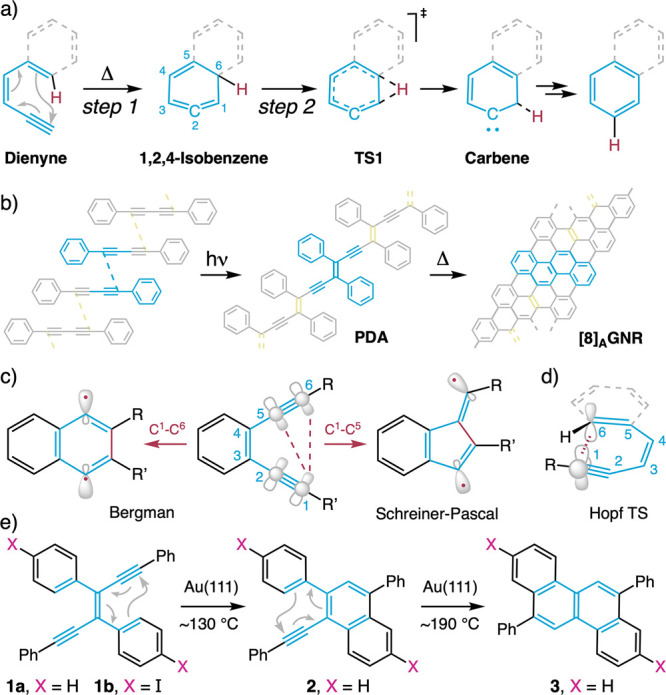
(a) Mechanism for the Hopf cyclization of a hexa-1,3-diene-5-yne (dienyne) to a new arene. (b) Illustration of the light-promoted topochemical polymerization of a diphenyl diacetylene into a polydiacetylene (**PDA**), and subsequent Hopf cyclization cascade and dehydrogenation to afford **[8]**_**A**_**GNR**. A chrysene subunit in **[8]**_**A**_**GNR** is shown in cyan color. (c) Two on-surface cyclization modes for *cis*-enediynes. (d) Illustration of the nonplanar transition state geometry in the Hopf 6π electrocyclization step. (e) Two consecutive Hopf cyclizations of enediyne **1a** to naphthalene **2** and chrysene **3**.

The Hopf cyclization stands out as one of the least exploited pericyclic reactions in organic synthesis (Figure 1a).^1−5^ It is an atom-neutral, thermally allowed 6π-electrocyclization that converts *cis*-hexa-1,3-diene-5-ynes and their benzannulated analogues into aromatic rings via two consecutive hydrogen shifts involving highly strained cyclohexa-1,2,4-triene (“1,2,4-isobenzene”) intermediates (Figure 1a).^2,6−11^ Its rate-determining step is not the initial 6π electrocyclization (step 1, Figure 1a), but the ensuing 1,2-H shift (step 2, **TS1**).^7,12−14^ While a photochemical variant has been described, its use has been limited to suitable scaffolds.^15−17^ The thermally induced Hopf cyclization has been disadvantaged by its high activation barrier, necessitating harsh reaction temperatures: >200–250 °C for nonbenzannulated dienynes^3,7,18^ and >300 °C for benzannulated dienynes.^7,12,13,18−20^

Despite these limitations, we have exploited the Hopf cyclization in reaction cascades that access previously unavailable armchair graphene nanoribbons such as [8]_A_GNR (Figure 1b).^12,13,19^ Arylated polydiacetylenes (e.g., PDA, Figure 1b), with their multiple embedded *cis-*hexa-1,3-diene-5-yne units, can be heated in the solid state at temperatures in excess of 330 °C to achieve full conversion to [8]_A_GNR,^12^ fjord-edge N_2_[8]GNR,^13^ and [12]_A_GNR.^19^ We show here that the temperature of the Hopf cyclization of model compounds **1a,b** can be lowered by nearly half upon heating on a surface of Au(111) (Figure 1e).

On-surface reactions over coinage metals have gained prominence for their extraordinary capacity to generate elaborate designs that are often not attainable via solution syntheses. This is by virtue of the requirements for rigorously clean ultrahigh vacuum conditions, as well as the surface confinement and catalytic effect that coinage metals exert on reaction mechanisms and their energy barriers.^21−33^ The combination of scanning-tunneling microscopy (STM) and noncontact atomic force microscopy (nc-AFM) enables visualization of intermediates in exquisite detail, as well as the role of the metal surface in coupling reactions.^33−38^

On-surface coupling reactions of alkyne substrates have included Glaser^30,33,39−48^ and Sonogashira reactions,^43,46,49^ dehalogenative or desilylative homocoupling of halo- or silylalkynes,^42,50−54^ decarboxylative coupling of alkynyl carboxylic acids,^55^ as well as various terminal alkyne coupling reactions generating enediynes or enetriynes,^56,57^ a [4]radialene via [1 + 1 + 1 + 1] coupling,^58^ or benzofulvenes via [2 + 2 + 1] coupling.^47,59^

**Figure 2:**
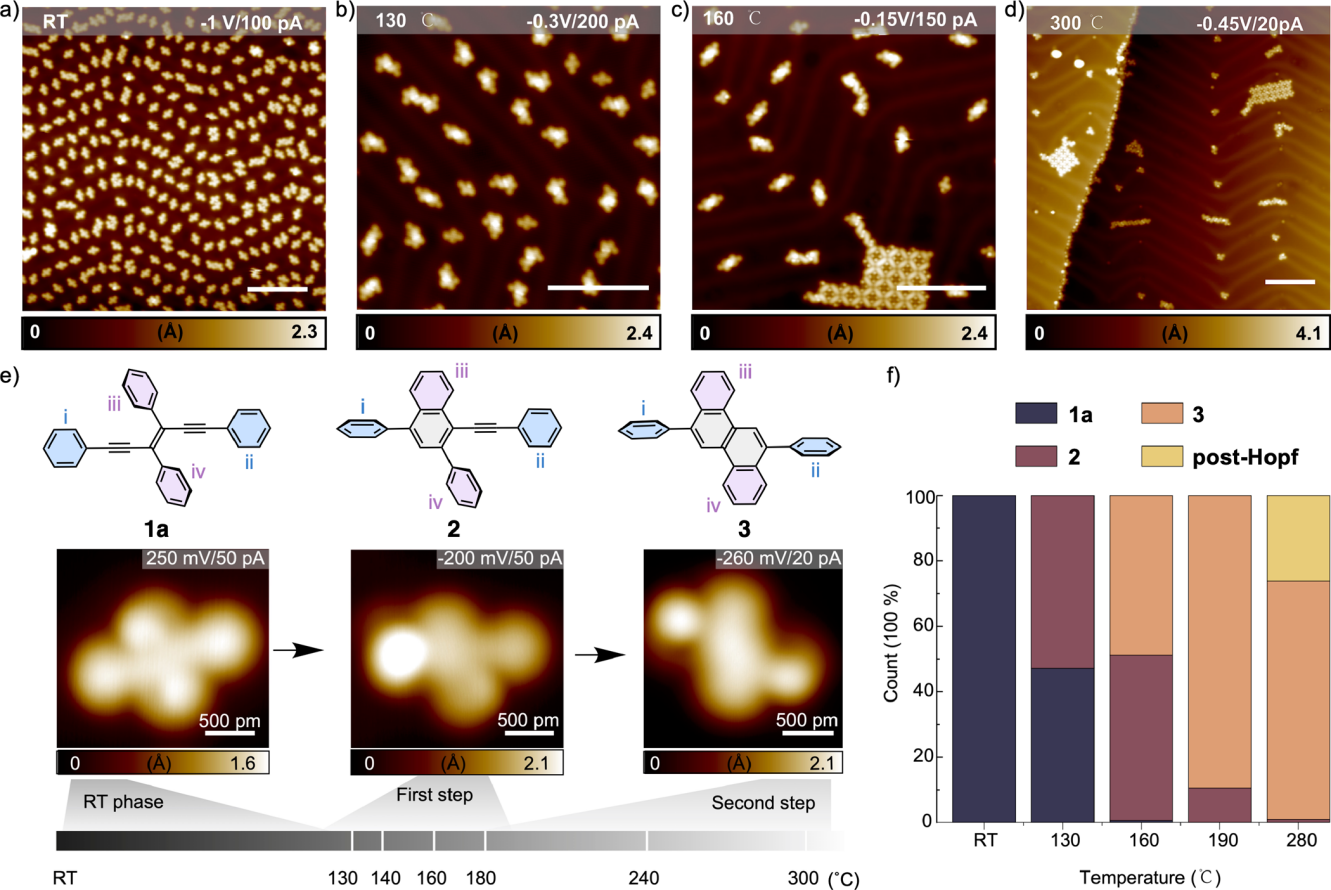
On-surface evolution of (*E*)*-*1,3,4,6-tetraphenyl-3-hexene-1,5-diyne (**1a**) during a step annealing sequence. Large-scale topographic images at (a) 20 °C (RT) and (b–d) after annealing for 15 min each at 130, 160, and 300 °C, respectively. White scale bars are 10 nm. (e) Structures with observed rotational isomerism (top) corresponding to the topographic images (middle) of **1a** (left), singly cyclized **2** (middle), and doubly cyclized **3** (right). The corresponding temperature ranges along which the reactions occur are shown at the bottom. (f) Bar chart of statistical distributions for **1a**, **2**, **3**, and post-Hopf species at different annealing temperatures.

To the best of our knowledge, an on-surface Hopf cyclization reaction has not been reported. Known on-surface, concerted cyclization reactions include the Bergman^60−635^ and alternate Schreiner-Pascal^64−61^ cyclization pathways of *cis*-enediynes (Figure 1c), as well as Huisgen “click” reactions,^68−70^ and an intramolecular hexadehydro-Diels–Alder reaction of a diyne-yne macrocycle.^72^ One should note that these concerted cyclizations take place exclusively via in-plane π-orbitals forming the new C–C bonds (e.g., Figure 1c, middle) and thus do not require partial molecule detachment from the surface of Au(111) at the transition state, which can be energetically costly. In the current study, the Hopf cyclization requires an out-of-plane approach between an alkenyl/aryl *p*-orbital at C^6^ and an alkynyl *p*-orbital at C^1^, Figure 1d), to form a C^1^–C^6^ bond, which is facilitated by the nonplanar conformation of the starting material **1a** on Au(111) (see below, Figure 2e, left) and the catalytic involvement of a gold atom (see the Calculations section).

In our previous work, the characterization of graphene nanoribbons was accomplished by solid-state CP-MAS ^13^C NMR, Raman, and IR spectroscopy on strongly aggregated bulk material, as well as by HR-TEM imaging.^12,13,19^ To gain better insight into the Hopf cyclization mechanism at the atomic level, it was clear that the structure of [8]_A_GNR would need to be reduced to one of its simplest constituents, chrysene (cyan subunit in Figure 1b, right). The latter would hence form by double Hopf cyclization of (*E*)-1,3,4,6-tetraphenyl-3-hexene-1,5-diyne (**1a**, Figure 1e).

**Figure 3:**
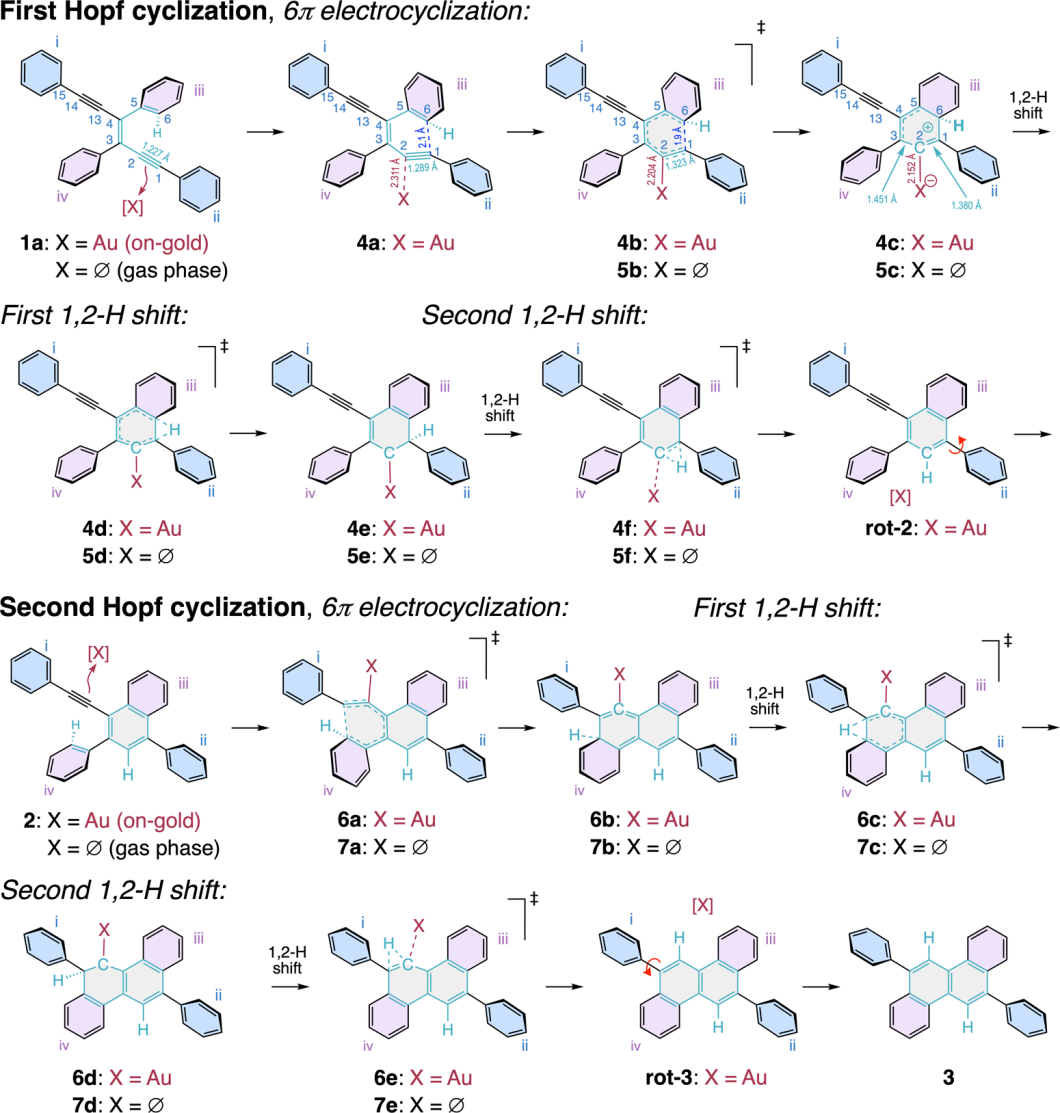
Mechanism of the Hopf cyclization of **1a** to afford naphthalene **2** and chrysene **3**, illustrated for a gold slab model and in the gas phase.

We describe here our initial results on enediyne **1a** and its corresponding polymer formed from the precursor, (*E*)-3,4-bis(4-iodophenyl)-1,6-diphenyl-3-hexene-1,5-diyne (**1b**), to reveal the reaction paths on Au(111) using STM and nc-AFM imaging. We find that the sequential Hopf cyclization of **1a** is completed entirely below 160 °C for the first cyclization, affording intermediate naphthalene derivative **2**, while the second cyclization, affording chrysene **3**, is completed by ∼190 °C (Figures 1e and 2e). The unexpected halving of the Hopf cyclization temperature, and its corresponding reaction barrier, compared to our bulk cyclization reactions^12,13,19^ is supported by DFT calculations. We find that a gold-alkyne interaction stabilizes intermediates in the Hopf cyclization on Au(111) (Figure 3), resulting in the lowering of the reaction barrier.

## Results and Discussion

We epitaxially deposited (*E*)-1,3,4,6-tetraphenyl-3-hexene-1,5-diyne (**1a**) by sublimation onto a Au(111) surface at 20 °C in an ultrahigh vacuum chamber with a base pressure of 1 × 10^–10^ mbar. The as-grown sample surface is shown in Figure 2a. Most molecules are isolated, with a preference to reside between the herringbone stripes of Au(111). All the molecules have *trans*-configuration.^73^ Enediyne **1a** has a nonplanar conformation on the surface of Au(111), with an inversion symmetry between the out-of-plane rotated four phenyl groups (Figure 2e, left).

To track all intermediates of the on-surface Hopf cyclizations, annealing was performed step by step at the temperatures shown in Figure 2e (bottom gray scale bar) with a 15 min stay at each temperature. After each annealing step, the sample was characterized in situ by a combination of STM and nc-AFM at 4.5 K. Surprisingly, the first Hopf cyclization, affording naphthalene derivative **2**, can already be observed after annealing at 130 °C (Figures 2b,f and S1). It is characterized by its apparent asymmetry in the topographic image (Figure 2e, middle). The phenyl group connected to the naphthyl ring is highly tilted and located significantly higher than the rest of the molecule, as revealed by its bright protrusion. As a result, monomers of **2** are not as “sticky” as **1a** on the gold surface and self-assemble into dimeric, trimeric, and tetrameric clusters away from uncyclized molecules of *trans*-enediyne **1a** (Figures 2b and S2a–e). Chrysene derivative **3** is not present anywhere on the surface, implying that the second Hopf cyclization has a substantially higher energy barrier. After annealing at 160 °C, we observe roughly half of the molecules becoming doubly cyclized with chrysene **3** (Figure 2c,f). As indicated by the topographic STM image (Figure 2e, right), the molecule is now nearly symmetric and exhibits an out-of-plane conformation of the two phenyl substituents (i) and (ii), with an inversion of symmetry between the tilted rings. Thus, chrysene **3** is also fairly mobile on the surface and forms self-assembled clusters, as revealed by the periodic chains and lattices shown in the topographic images (Figures 2c and S2f–h). Finally, higher-temperature annealing at 300 °C generates 5-membered-annulated species, named post-Hopf (Figures 2d,f and S3), which involves a well-known dehydrocyclization process at higher temperatures on Au(111).^23^ Statistical analysis of the molecular distributions at 20, 130, 160, 190, and 280 °C, respectively, reflects the evolution of ratios between structures **1a**, **2**, and **3** (Figure 2f). Most of the molecules undergo single Hopf cyclization below 160 °C, and double Hopf cyclization between 160 and 200 °C.

### Calculations

To compare the gas phase and on-surface mechanisms, DFT calculations were carried out on the conversion of enediyne **1a** to naphthalene **2** and chrysene **3**, both in the gas phase and on a Au(111) slab model (Table 1, Figure 3).^73^ In the gas phase, the intermediates in the Hopf cyclization calculations were analogous to those previously described by Prall et al. and our own work.^7,12,13^ More specifically, the Hopf cyclization steps were verified to proceed via an initial 6π-electrocyclization, followed by two consecutive 1,2-H shifts, where the first H-shift was the rate-determining step (Table 1, Figure 3). All gas phase structures were optimized at the B3LYP-D3/6-31G(d) level, with D3 dispersion correction applied to account for steric interactions, and single-point energy calculations were performed at the M06-2*X*/6-311+G(d,p) level of theory, with B3LYP-D3/6-31G(d) frequency calculations to obtain the free energy values (see Table 1).^73^

**Table 1:** Calculated Relative Free Energies of the Intermediates in the Hopf Cyclization of Enediyne **1a** to Naphthalene **2** and Chrysene **3** on a Au(111) Slab Model and in the Gas Phase

compound		relative free energy^a^^,^^b^			
Au(111)	gas phase	Au(111)		gas phase	
		rel. to 1a	rel. to 2	rel. to 1a	rel. to 2
**1a**		0.0		0.0	
**4b**	**5b**	**28.6**		**52.3**	
**4c**	**5c**	23.1		45.3	
**4d**	**5d**	**32.3**^c^		**59.7**^c^	
**4e**	**5e**	9.5		29.6	
**4f**	**5f**	**23.1**		**39.3**	
**2**		–54.7	0.0	–50.7	0.0
**6a**	**7a**	***–20.8***	**33.9**	**5.3**	**56.0**
**6b**	**7b**	–23.1	31.6	1.6	52.3
**6c**	**7c**	***–15.0***^c^	**39.7**	**9.5**^c^	**60.2**^c^
**6d**	**7d**	–37.4	17.3	–20.8	29.9
**6e**	**7e**	***–23.1***	**31.6**	***–14.9***	**35.8**
**3**		–100.6	–45.9	–95.0	–44.3

a Free energy values are in kcal/mol.

b Values shown in bold italic are the transition states.

c Rate-determining barrier.

For the on-surface calculations, all structures were computed at the PBE level of theory,^73,74^ with D3 dispersion correction also applied, and with electronic states expanded using a TZV2P contracted Gaussian basis set for carbon and hydrogen and a DZVP basis set for gold. Transition states were assessed by performing, for each reaction, a sequence of constrained geometry optimizations that identified a minimum energy path (MEP). A reaction coordinate was defined and constrained to a value that was changed in small increments at each subsequent optimization. The highest energy structures in each MEP were taken as approximations for the corresponding transition states for each of the reaction steps. The Au(111) surface was modeled with a periodic slab geometry constructed from a four-layer gold supercell with a top surface size of 38.3 × 32.4 Å^2^ (768 gold atoms). The on-surface reaction steps using this gold slab proceeded generally in parallel to those in the gas phase (Table 1, Figures 3, S5), namely, through the initial 6π-electrocyclization followed by the two consecutive 1,2-H shifts. However, an essential difference was that most of the steps involved unforeseen bonding to a gold atom. The latter proved to be key to explaining the dramatic acceleration of the Hopf cyclization on Au(111).

**Figure 4:**
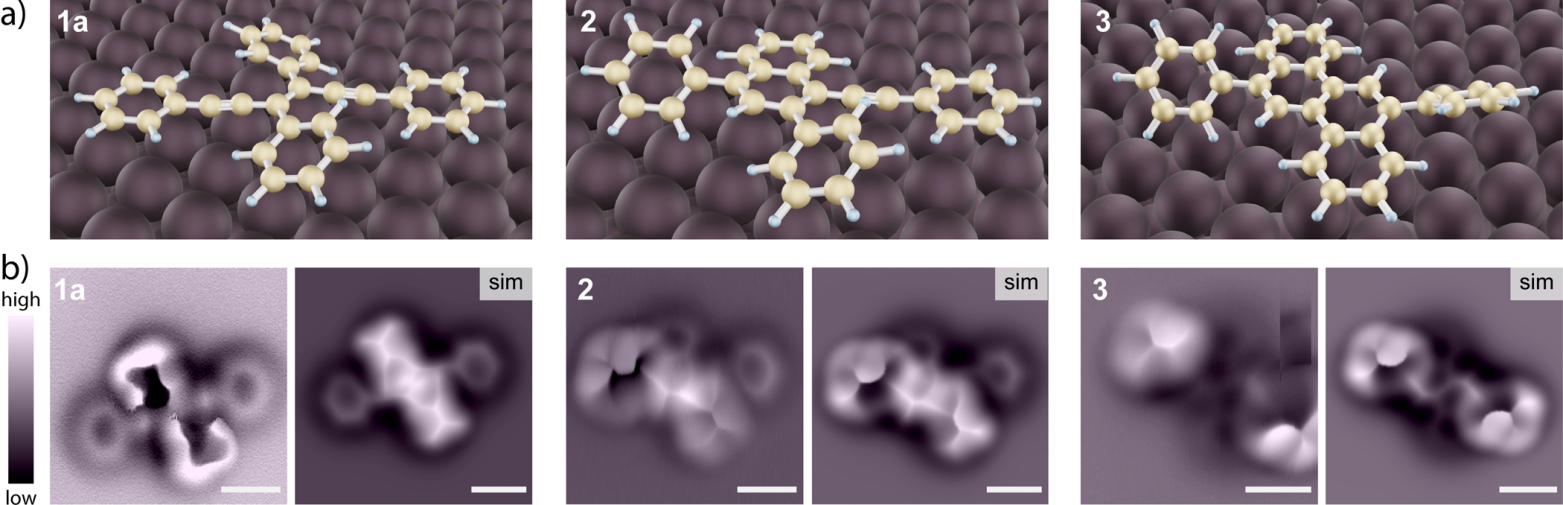
(a) DFT optimized ground-state geometries for **1a**, **2**, and **3** on a Au(111) slab model. (b) In the same order, experimentally observed nc-AFM images (left) and corresponding simulated AFM images (right). White scale bars are 0.5 nm.

We first confirmed the accuracy of our on-surface calculations by reproducing the adsorptional geometries of experimentally observed structures **1a**, **2**, and **3** (Figure 4). The optimized geometry of enediyne **1a** on Au(111) has its two phenyl groups (i) and (ii) nearly coplanar with the gold surface, while phenyl groups (iii) and (iv) are tilted out-of-plane due to steric interactions with the rest of the molecule (Figure 4a). Thus, the conformation of **1a** is the same as that in Figure 2e, left. A simulated AFM image using this ground-state geometry is compared to the experimentally observed nc-AFM image of **1a** (Figure 4b, left), showing good consistency between the two. A small difference is that this experimental image does not simultaneously show the flat-lying (*E*)-1,6-diphenyl-3-hexene-1,5-diyne moiety and its tilted 2,3-diphenyl substituents ((iii) and (iv) in Figure 2e). However, a series of nc-AFM images using a range of tip-molecule distances confirms all the features in the simulated images of the molecule (Figure S4). Similarly, naphthalene **2** and chrysene **3** are also well replicated by our on-surface optimizations, confirmed by the uniformity between simulated and experimental nc-AFM images (Figure 4b, middle and right).

### On-Surface Reaction Mechanism and Involvement of a Gold Atom

Calculated geometries, relative free energies, and a comparison of the progression of the Hopf cyclization along the reaction coordinate in the gas phase and on-surface are provided in Table 1 and Figures 3 and S5. Geometries, bond lengths, and angles of selected intermediates obtained in MEP calculations for the first 6π-electrocyclization step are shown in Figure S6A. The calculations reveal an unexpected turn in the reaction mechanism, one involving the participation of a gold atom from the Au(111) surface in the ring-forming and 1,2-H shift reaction steps (Figures 3 and S6A). This participation plays a crucial role in lowering the reaction barrier, switching from the anticipated high-barrier 6π*-*electrocyclization mechanism to the much lower-barrier Au-catalyzed cyclization.

**Figure 5:**
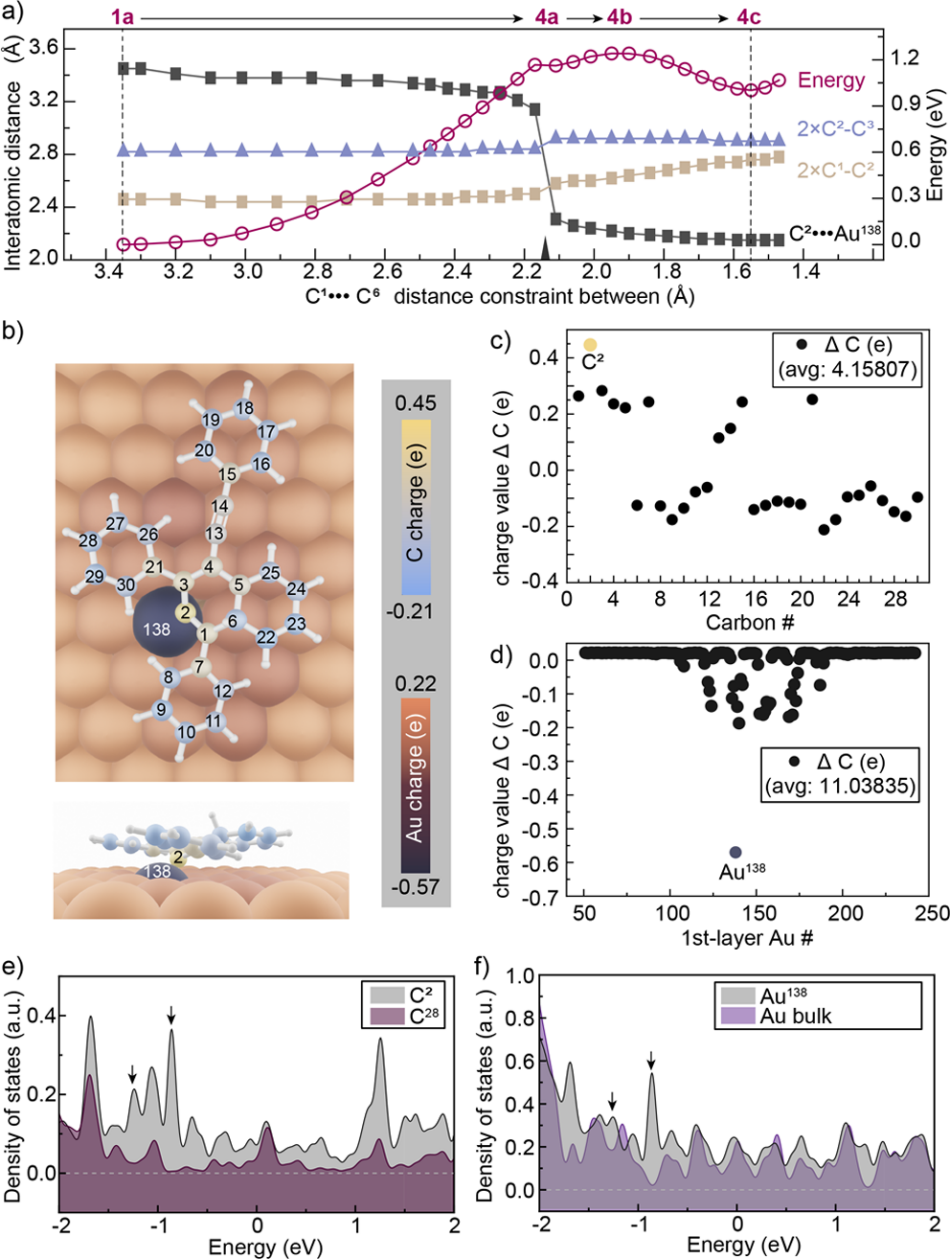
MEP calculation of the first 6π-electrocyclization steps and Hirshfeld charge analysis. (a) Changes in the bond length (Å) between C^2^–Au^138^ (dark gray squares), C^1^–C^2^ (golden brown squares), C^2^–C^3^ (mauve triangles), and free energies (eV) at each step of the MEP calculation starting from enediyne **1a**, forming gold-allene complex **4c** via the transition state **4b**. Bond lengths for C^1^–C^2^ and C^2^–C^3^ are plotted twice their value for clarity. (b) Top view of the geometry of cyclization intermediate **4c** calculated at the PBE level of theory on a gold slab (top); side view with Au^138^ detaching from the surface of the gold slab (bottom). The color-coded spatially resolved charge values for carbons C^1^–C^30^ and Au atoms obtained from a Hirshfeld charge population analysis are also shown. (c, d) Charge deviation from the average population (avg) for carbon and first-layer Au atoms. The avg is 4.01773 electrons for carbon and 11.03194 for first-layer gold. (e) Calculated partial density of states (PDOS) for carbon atom C^2^ (gray) and all others (maroon); arrows indicate new states for carbon atom C^2^. (f) Calculated PDOS for gold surface atom Au^138^ (gray) and bulk gold atoms (mauve); arrows imply new states for the gold surface atom Au^138^ that has formed a new bond to C^2^.

To understand how the potential catalytic effect of the gold surface could take place, we first calculated how the structure of enediyne **1a** changes during the 6π-electrocyclization step, as the distance between carbon atoms C^1^ and C^6^ is progressively restricted to shorter values (Figure 5 and S6A). The energy for each stepwise C^1^···C^6^ distance constriction to intermediate **4c** is shown in purple circles in Figure 5a. Gold-catalysis begins upon constriction of the C^1^···C^6^ distance in the MEP calculations, upon which carbon C^2^ of one of the C≡C bonds in enediyne **1a** starts interacting with the gold atom Au^138^. Upon further constriction of the C^1^···C^6^ distance from 2.2 to 2.1 Å, a sudden contraction of the Au^138^···C^2^ distance occurs, changing from 3.145 to 2.311 Å. At this stage, a formal gold–carbon bond has essentially formed, represented by intermediate structure **4a** (Figures 3, 5a, and S6A). However, this intermediate structure is not yet a transition state but can be viewed as a transient gold-alkyne complex whose C^1^≡C^2^ alkyne moiety has reached substantial bonding through bending (∠C^1^–C^2^–C^3^ = 133.0°, ∠C^3^–C^2^–C^20^ = 142.7°). For reference, several crystal structures of gold(I) η^2^-complexes with open-chain or cyclic alkynes have been reported.^75^ The C≡C bond lengths in these complexes range from 1.220 to 1.233 Å,^76^ and alkyne bending angles range from 155.0 to 168.1°.^77^

Further constriction of the C^1^···C^6^ distance to 1.9 Å results in the highest energy intermediate in the 6π-electrocyclization step, labeled **4b**, which we define as the transitions state (Figures 3, 5a, and S6A). The C^1^···C^6^ distance in **4b** is slightly longer, by 0.1 Å, to that found (1.801 Å) for the analogous calculated transition state **5b** in the gas phase calculations (Figure 3). Additionally, the Au^138^···C^2^ distance has shortened further to 2.204 Å from that in **4a**. The barrier for this first 6π*-*electrocyclization is 28.6 kcal/mol, which is nearly half (55%) of that calculated for the gas phase (52.3 kcal/mol, Table 1).

Final constriction of the C^1^···C^6^ distance arrives at the strained gold allene η^1^-complex **4c**, which is a local minimum (Figures 3, 5a, and S6A). Interestingly, the C^1^–C^2^ bond length, at 1.380 Å, is now close to that of a regular C=C double bond (1.34 Å), while the C^2^–C^3^ bond length, at 1.451 Å, resembles that of a conjugated sp^2^C–sp^2^C single bond (1.47 Å).^78^ In addition, the C^1^–C^2^ bond is now strongly bent (∠C^1^–C^2^–C^3^ = 126.5°, ∠C^2^–C^1^–C^20^ = 133.1°). As the C^1^≡C^2^ alkyne moiety of intermediate **4a** has been converted into a C=C bond in allene complex **4c**, the Au^138^···C^2^ distance has further reduced to 2.152 Å.

The calculated structure of **4c** is intriguing in that it resembles two known gold(I) allene η^1^-complexes (**8** and **9**, Figure S7), which are very similar in geometry despite the +1 gold oxidation state for these complexes. Stephan and co-workers have reported the synthesis and complexation of tetraphenyl carbodicyclopropenylidene, an all-carbon carbidodicarbene, with an *N*-heterocyclic carbene (NHC) gold(I) species (**8**, Figure S7),^79^ and Fürstner et al. have described the complexation of tetrakis(dimethylamino)allene with Ph_3_PAuCl to give another stable carbidodicarbene complex (**9**),^80^ both characterized by X-ray diffraction. The two complexes have identical gold(I)–carbon distances at 2.071 Å, which are comparable to the length calculated for gold(0) complex **4c** (2.152 Å). The allene C=C bonds in Stephan’s carbidodicarbene complex are 1.372 and 1.362 Å, which compare well with that of the C^1^=C^2^ bond in **4c** (1.380 Å). In addition, the C^2^–C^3^ bond length (1.451 Å) is similar to the allene bond lengths in bis-NHC carbidodicarbene **9** (1.407 and 1.424 Å); these bonds have single bond character due to the π-delocalizing effect of the adjacent dimethylamino groups, as shown in the polar resonance structure of **9** (Figure S7). Intriguingly, the lengths of the C^3^–C^4^ and C^4^–C^5^ bonds in intermediate **4c** are 1.426 and 1.432 Å, respectively (Figure 3). These bonds can therefore be considered a delocalized allylic system, as similar C–C bond lengths in the crystal structures of three different uncomplexed allylic cations range from 1.36 to 1.43 Å (Figure S8),^81^ while the neutral allyl radical C_3_H_5_· in the gas phase has a bond length of 1.428 Å.^82^ With these considerations, we assign intermediate **4c** the delocalized zwitterionic structure shown in Figure 3, whose gold atom Au^138^ is partially negatively charged, while the balancing positive charge for this overall neutral species is delocalized between C^2^ and C^3^–C^4^–C^5^ in the form of an allylic cation.

Alabugin has analyzed a similar effect observed for the in-solution gold(I)-catalyzed Bergman cyclization of *cis*-enediynes, where gold promotes crossover from the classical diradical mechanism into a zwitterionic one.^83−86^ Upon coordination of a gold atom to one of the triple bonds of a *cis*-enediyne, the mechanism becomes exergonic, and the positive charge of the zwitterionic form of *p*-benzyne gets stabilized dramatically to afford an intermediate that is the global minimum on the potential energy surface. Correspondingly, our Hopf cyclization of **1a** on Au(111) is likely also electrophilic in nature, and we are currently exploring this exciting prospect for increasing the rate of Hopf cyclizations in solution.

A Hirshfeld charge population analysis, which yields chemically meaningful charges,^87^ was performed on gold allene complex **4c** to support this assignment (Figure 5b–d). The charge values for the carbon and gold atoms of **4c** are plotted as spatially resolved charge deviations from the average value (Figure 5b–d). Carbon atom C^2^ has the most charge deviation (positive) from the average value (Figure 5c) and is the one formally bonded to the gold surface. Correspondingly, the gold atom Au^138^, adjacent to C^2^, has an opposite, negative charge (Figure 5d), reinforcing the case for the formation of a polar bond between these two atoms. Furthermore, the projected density of states (PDOSs) of individual atoms, shown in Figure 5e,f, adds further support for the formation of a polar Au–C bond between C^2^ and Au^138^. This is revealed by the emergence of new electronic states marked by black arrows. Overall our analysis strongly supports the formation of a Au–C bond, which is essential in lowering the barrier of the on-surface Hopf cyclization.

The next step of the on-surface reaction, which is rate-determining for the overall enediyne-to-naphthalene transformation (**1a**–**2**), is the first of two 1,2-H migrations, affording the gold-complexed carbene intermediate **4e** (Figures 3 and S6B). The barrier (32.3 kcal/mol) for this step is transition state **4d**, whose structure parallels that of the gas phase, but for which C^2^ remains bound to Au^138^. Again, bonding to gold appears to contribute significantly to halving (54%) the overall reaction barrier from 59.7 kcal/mol in the gas phase down to 32.3 kcal/mol. The involvement of Au^138^ in the transition state for the first 1,2-H shift **4d**, its carbene product **4e**, and the transition state for the second 1,2-H shift **4f** is clearly visible in Figure S6B. Gold stays bound to C^2^ in all intermediates **4d**–**f**, with the Au^138^–C^2^ bond length varying between 2.07 and 2.17 Å. Hirshfeld charge population analysis of the transitions state structures **4d** and **4f** again supports the electrophilic nature of the overall rearrangement, with C^2^ being most positively charged, and Au^138^ negatively charged (Figure S11).

The final steps of the Hopf cyclization on the gold slab model proceed through a second, gold-bound 1,2-H shift, giving initially rotamer **rot-2** (−48.9 kcal/mol in Figure 3, value not shown in Table 1). Then, the naphthalene-bound phenyl ring ii rotates to give the final product **2** (−50.7 kcal/mol), which is 1.8 kcal/mol lower in energy.

For the second Hopf cyclization sequence (Table 1, Figure 3), gold-bound intermediates **6a**–**e** have geometries similar to those of the species calculated in the first cyclization sequence. This sequence provides a second rate-determining barrier for this reaction, embodied by transition state **6c**. The reaction barrier, at −15.0 kcal/mol (+39.7 kcal/mol relative to **2**), is 7.4 kcal/mol higher than the barrier for the first cyclization sequence (+32.3 kcal/mol). We attribute this increase in the reaction barrier to the presence of the additional naphthyl ring obtained during the first Hopf cyclization, whose aromaticity needs to be broken during the 6π-electrocyclization and first 1,2-H shift steps.

Interestingly, during our heating experiments with enediyne **1a** on Au(111), we observed an inconsequential number of pentafulvene molecules **2′** by STM, out of hundreds of molecules of naphthalene derivative **2** (Figure S9). This observation shows that the alternate Schreiner-Pascal cyclization pathway between C^2^ and C^6^ can also occur (Figure 1c),^66,67^ but with a much lower probability, as underscored by the relative calculated reaction barriers for both pathways pictured in Figure S9.

### Synthesis of Novel Graphene Nanoribbons

**Figure 6:**
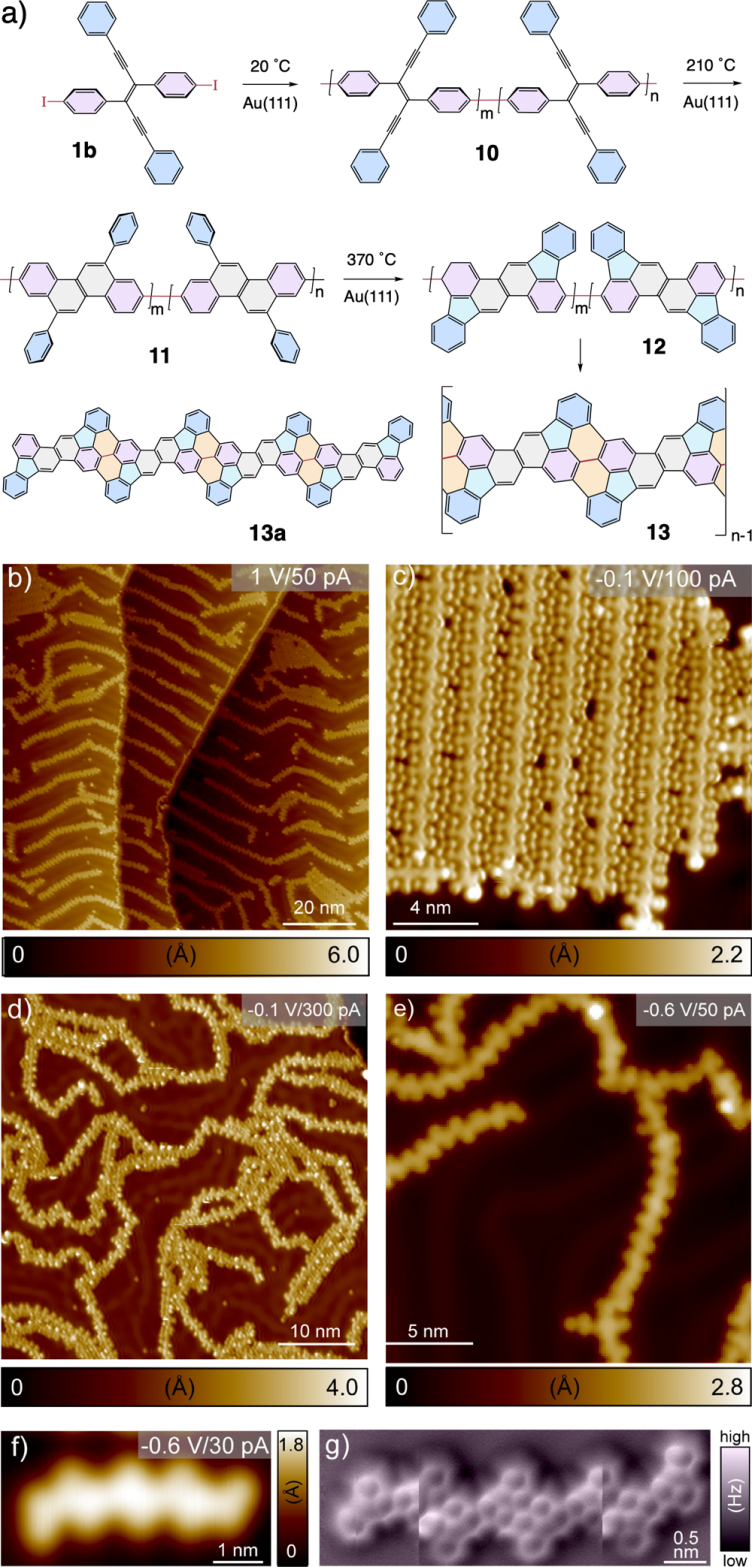
(a) Reaction scheme for the multifold Ullmann-like coupling of (*E*)-3,4-bis(4-iodophenyl)-1,6-diphenyl-3-hexen-1,5-diyne (**1b**) on a Au(111) surface at 20 °C, affording polymer **10**, followed by a cascade of Hopf cyclizations (**11**) and further graphitization to graphene nanoribbons **12**, and then **13**. (b, c) Overview STM topographic images of the polymer strands (**10**) obtained after depositing diiodo enediyne **1b** on a Au(111) surface at 20 °C. Both cisoid and transoid arrangements between individual enediyne units within **10** can be clearly seen in (c). (d, e) Overview STM topographic images after annealing **10** for 20 min at 210 and 370 °C, respectively, affording first **11** then **12** and **13**. (f, g) STM topography (f) and nc-AFM (g) images of nanoribbon oligomer **13a** , represented by its matching chemical structure in (a).

With the promising results obtained on enediyne **1a**, we wanted to demonstrate that the Hopf cyclization can be advantageously exploited to synthesize isolated graphene nanoribbons on a Au(111) surface at moderate temperatures, allowing characterization of the products by tip imaging techniques, as well as providing validation for our bulk GNR synthesis method which exploits the Hopf cyclization.^12,13,19^ A straightforward way to investigate this path was to bis-halogenate structure **1a** with either bromine or iodine substituents and then polymerize the resulting monomers into 1D chains through Ullmann-like coupling on Au(111). We wanted a system that would undergo 1D-polymerization prior to the Hopf cyclization sequence. Thus, iodination, exemplified by diiodo enediyne **1b**, was adopted owing to the facile Ullmann-like coupling propensity of C–I bonds on Au(111), which can occur as low as ambient temperature.^88^ The set of reactions starting from diiodo enediyne **1b** is shown in Figure 6a. We deposited diiodo enediyne **1b** on a Au(111) surface under the same conditions as those for enediyne **1a**. However, unlike the isolated molecules of enediyne **1a**, we did not see any free molecules of diiodo enediyne **1b**; instead, they were already coupled into the 1D-polymeric chains **10** at 20 °C (Figure 6b,c). Isolated iodine atoms can be seen moving freely on the Au(111) surface, with a strong preference for locating between aggregates of the polymeric enediyne chains of **10**. The formation of cisoid and transoid connections between individual enediyne units is also clearly visible in Figure 6c and over a larger area STM image in Figure S12a. Following the same heating protocol carried out on enediyne **1a**, we obtained chains of poly(chrysene) **11**, clearly showing the chrysene units in a periodically repeating pattern, albeit with many kinks that we attribute to ring fusions deriving from the penultimate enediyne units of **10** in a cisoid relationship (Figure 6d). After annealing at 370 °C, most of the chains are converted into 5-membered-annulated species (**12**) and further annealed aromatic ribbons with structure **13**. While the structure of **13** is quite complex due to its cisoid/transoid distribution of monomeric units (Figures 6e and S12d), many sections have longer runs of transoid regioisomers that can be clearly seen, for example on the right side of Figure 6e. Figure 6f gives an example of an isolated oligomeric nanoribbon obtained at 370 °C, whose nc-AFM image (Figure 6g) is in good agreement with the structural features of **13a** displayed in Figure 6a. Because the all-transoid form of graphene nanoribbon **13** has unusual 5-membered ring fusions, we have calculated its band structure as well as the projected density of states (Figure S10). The band structure of **13** indicates that it has a bandgap of ∼3 eV (left panel), making it a wide-bandgap semiconductor.

## Conclusion

In summary, we have shown that the reaction barrier of the Hopf cyclization can be significantly lowered on a Au(111) surface for two model systems, (*E*)-1,3,4,6-tetraphenyl-3-hexene-1,5-diyne (**1a**) and (*E*)-3,4-bis(4-iodophenyl)-1,6-diphenyl-3-hexene-1,5-diyne (**1b**). We have used scanning tunneling microscopy (STM) and noncontact atomic force microscopy (nc-AFM) to monitor the two sequential Hopf cyclizations of enediynes **1a,b** on Au(111). The experimental results and DFT calculations show that the reaction barrier is effectively halved through the involvement of a gold atom on the Au(111) surface. These results show that, in principle, a large diversity of graphene nanoribbons may be obtainable through a combination of Ullmann-like and Hopf cyclization reactions on Au(111). The involvement of gold catalysis in the reaction mechanism opens a new avenue for creating novel nanographene structures, both in solution and on Au(111), which we are currently pursuing.

## Data Availability

Data related to the simulations will be available on the Materials Cloud repository at DOI: 10.24435/materialscloud:62-ew.

## Supporting Information Available

Experimental and computational methods, supporting STM and nc-AFM data, calculations, detailed synthesis description of chemical compounds reported in this study, and crystallographic data for enediynes **1a** and **1b**(89) (PDF)

## References

[ref1] Hopf H.; Musso H. Preparation of Benzene by Pyrolysis of *cis*- and *trans*-1,3-Hexadien-5-yne. Angew. Chem., Int. Ed. Engl. 1969, 8, 680–680. 10.1002/anie.196906801.

[ref2] Hopf H.; Berger H.; Zimmermann G.; Nüchter U.; Jones P. G.; Dix I. Formation of Isobenzenes by Thermal Isomerization of 1,3-Hexadiene-5-yne Derivatives. Angew. Chem., Int. Ed. Engl. 1997, 36, 1187–1190. 10.1002/anie.199711871.

[ref3] Roth W. R.; Hopf H.; Horn C. 1,3,5-cyclohexatrien-1,4-diyl und 2,4-cyclohexadien-1,4-diyl. Chem. Ber. 1994, 127, 1765–1779. 10.1002/cber.19941270929.

[ref4] Kobayashi S.; Jo̷rgensen K. A., Eds. Cycloaddition Reactions in Organic Synthesis; Wiley-VCH: Weinheim, 2002.

[ref5] Vogel P.; Houk K. N.; Vogel P.; Houk K. N. Organic Chemistry: Theory, Reactivity and Mechanisms in Modern Synthesis; Wiley, 2019; pp. 349–614.

[ref6] Christl M.; Groetsch S. Cycloallenes. Part 13. Cyclohexa-1,2,4-triene from 1-Bromocyclohexa-1,4-diene. Eur. J. Org. Chem. 2000, 1841–2001. 10.1002/(sici)1099-0690(200005)2000:10<1871::aid-ejoc1871>3.0.co;2-3.

[ref7] Prall M.; Krüger A.; Schreiner P. R.; Hopf H. The Cyclization of Parent and Cyclic Hexa-1,3-dien-5-ynes—A Combined Theoretical and Experimental Study. Chem.—Eur. J. 2001, 7 (20), 4386–4394. 10.1002/1521-3765(20011015)7:20<4386::AID-CHEM4386>3.0.CO;2-S. 11695672

[ref8] Hopf H.; Krüger A. Synthesis of Cyclo-1,3-dien-5-ynes. Chem.—Eur. J. 2001, 7, 4378–4385. 10.1002/1521-3765(20011015)7:20<4378::AID-CHEM4378>3.0.CO;2-I. 11695671

[ref9] Christl M.; Braun M.; Müller G. 1,2,4-Cyclohexatriene, an Isobenzene, and Bicyclo[4.4.0]Deca-1,3,5,7,8-pentaene, an Isonaphthalene: Generation and Trapping Reactions. Angew. Chem., Int. Ed. Engl. 1992, 31, 473–476. 10.1002/anie.199204731.

[ref10] aShakespeare W. C.; Johnson R. P. 1,2,3-Cyclohexatriene and Cyclohexen-3-yne: Two New Highly Strained C_6_H_6_ Isomers. J. Am. Chem. Soc. 1990, 112, 8578–8579. 10.1021/ja00179a050.

[ref11] Kaplan L.; Walch S. P.; Wilzbach K. E. Photolysis of Benzene Vapor at 1849 Å. Formation of cis-1,3-Hexadien-5-yne. J. Am. Chem. Soc. 1968, 90, 5646–5647. 10.1021/ja01022a081.

[ref12] Jordan R. S.; Li Y. L.; Lin C.-W.; McCurdy R. D.; Lin J. B.; Brosmer J. L.; Marsh K. L.; Khan S. I.; Houk K. N.; Kaner R. B.; Rubin Y. Synthesis of *N* = 8 Armchair Graphene Nanoribbons from Four Distinct Polydiacetylenes. J. Am. Chem. Soc. 2017, 139, 15878–15890. 10.1021/jacs.7b08800. 29083160

[ref13] Li Y. L.; Zee C.-T.; Lin J. B.; Basile V. M.; Muni M.; Flores M. D.; Munárriz J.; Kaner R. B.; Alexandrova A. N.; Houk K. N.; Tolbert S. H.; Rubin Y. Fjord-Edge Graphene Nanoribbons with Site-Specific Nitrogen Substitution. J. Am. Chem. Soc. 2020, 142, 18093–18102. 10.1021/jacs.0c07657. 32894950

[ref14] Xu Q.; Hoye T. R. A Distinct Mode of Strain-Driven Cyclic Allene Reactivity: Group Migration to the Central Allene Carbon Atom. J. Am. Chem. Soc. 2023, 145, 9867–9875. 10.1021/jacs.3c02469. 37086185 PMC10864128

[ref15] Op den Brouw P. M.; Laarhoven W. H. The Photochemistry of 2-Vinyldiphenylacetylene and Related Compounds. J. Chem. Soc., Perkin Trans. 1982, 795–799. 10.1039/p29820000795.

[ref16] Sajimon M. C.; Lewis F. D. Photocyclization of 2-Vinyldiphenylacetylenes and Behavior of the Isonaphthalene Intermediates. Photochem. Photobiol. Sci. 2005, 4, 629–636. 10.1039/b504997k. 16052270

[ref17] Hitt D. M.; O’Connor J. M. Acceleration of Conjugated Dienyne Cycloaromatization. Chem. Rev. 2011, 111, 7904–7922. 10.1021/cr2001542. 21978178

[ref18] Zimmermann G. Cycloaromatization of Open and Masked 1,3-Hexadien-5-ynes – Mechanistic and Synthetic Aspects. Eur. J. Org. Chem. 2001, 457–471. 10.1002/1099-0690(200102)2001:3<457::aid-ejoc457>3.0.co;2-b.

[ref19] Jordan R. S.; Wang Y.; McCurdy R. D.; Yeung M. T.; Marsh K. L.; Khan S. I.; Kaner R. B.; Rubin Y. Synthesis of Graphene Nanoribbons via the Topochemical Polymerization and Subsequent Aromatization of a Diacetylene Precursor. Chem. 2016, 1, 78–90. 10.1016/j.chempr.2016.06.010.

[ref20] Nüchter U.; Zimmermann G.; Francke V.; Hopf H. Thermal Rearrangements, 28. Competing Reaction Pathways in the Thermal Cycloisomerization of 1,3-Hexadien-5-ynes. Liebigs Ann. 1997, 1997, 1505–1515. 10.1002/jlac.199719970729.

[ref21] Grill L.; Dyer M.; Lafferentz L.; Persson M.; Peters M. V.; Hecht S. Nano-Architectures by Covalent Assembly of Molecular Building Blocks. Nat. Nanotechnol. 2007, 2, 687–691. 10.1038/nnano.2007.346. 18654406

[ref22] Cai J.; Ruffieux P.; Jaafar R.; Bieri M.; Braun T.; Blankenburg S.; Muoth M.; Seitsonen A. P.; Saleh M.; Feng X.; Müllen K.; Fasel R. Atomically Precise Bottom-up Fabrication of Graphene Nanoribbons. Nature 2010, 466, 470–473. 10.1038/nature09211. 20651687

[ref23] Ruffieux P.; Wang S.; Yang B.; Sánchez-Sánchez C.; Liu J.; Dienel T.; Talirz L.; Shinde P.; Pignedoli C. A.; Passerone D.; Dumslaff T.; Feng X.; Müllen K.; Fasel R. On-Surface Synthesis of Graphene Nanoribbons with Zigzag Edge Topology. Nature 2016, 531, 489–492. 10.1038/nature17151. 27008967

[ref24] Mishra S.; Catarina G.; Wu F.; Ortiz R.; Jacob D.; Eimre K.; Ma J.; Pignedoli C. A.; Feng X.; Ruffieux P.; Fernández-Rossier J.; Fasel R. Observation of Fractional Edge Excitations in Nanographene Spin Chains. Nature 2021, 598, 287–292. 10.1038/s41586-021-03842-3. 34645998

[ref25] Held P. A.; Fuchs H.; Studer A. Covalent-Bond Formation via On-Surface Chemistry. Chem.—Eur. J. 2017, 23, 5874–5892. 10.1002/chem.201604047. 28097707

[ref26] Wang C.; Zhang H.; Chi L. Covalently Bonded Organic Structures via On-Surface Synthesis. In Supramolecular Chemistry on Surfaces, Champness N., Ed.; Wiley, 2002, pp. 135–169.

[ref27] Treier M.; Pignedoli C.; Laino T.; Rieger R.; Müllen K.; Passerone D.; Fasel R. Surface-Assisted Cyclodehydrogenation Provides a Synthetic Route towards Easily Processable and Chemically Tailored Nanographenes. Nat. Chem. 2011, 3, 61–67. 10.1038/nchem.891. 21160519

[ref28] Saywell A.; Schwarz J.; Hecht S.; Grill L. Polymerization on Stepped Surfaces: Alignment of Polymers and Identification of Catalytic Sites. Angew. Chem., Int. Ed. 2012, 51, 5096–5100. 10.1002/anie.201200543. 22522422

[ref29] Lackinger M. Surface-Assisted Ullmann Coupling. Chem. Commun. 2017, 53, 7872–7885. 10.1039/C7CC03402D. 28649683

[ref30] Liu W.; Luo X.; Bao Y.; Liu Y. P.; Ning G.-H.; Abdelwahab I.; Li L.; Nai C. T.; Hu Z. G.; Zhao D.; Liu B.; Quek S. Y.; Loh K. P. A Two-Dimensional Conjugated Aromatic Polymer via C–C Coupling Reaction. Nat. Chem. 2017, 9, 563–570. 10.1038/nchem.2696. 28537590

[ref31] Chen H.; Zhu H.; Huang Z.; Rong W.; Wu K. Two-Sidedness of Surface Reaction Mediation. Adv. Mater. 2019, 31, 1902080 10.1002/adma.201902080. 31418920

[ref32] Fritton M.; Duncan D. A.; Deimel P. S.; Rastgoo-Lahrood A.; Allegretti F.; Barth J. V.; Heckl W. M.; Björk J.; Lackinger M. The Role of Kinetics versus Thermodynamics in Surface-Assisted Ullmann Coupling on Gold and Silver Surfaces. J. Am. Chem. Soc. 2019, 141, 4824–4832. 10.1021/jacs.8b11473. 30817138

[ref33] Wang T.; Zhu J. Confined On-Surface Organic Synthesis: Strategies and Mechanisms. Surf. Sci. Rep. 2019, 74, 97–140. 10.1016/j.surfrep.2019.05.001.

[ref34] Shen Q.; Gao H.-Y.; Fuchs H. Frontiers of On-Surface Synthesis: From Principles to Applications. Nano Today 2017, 13, 77–96. 10.1016/j.nantod.2017.02.007.

[ref35] Clair S.; de Oteyza D. G. Controlling a Chemical Coupling Reaction on a Surface: Tools and Strategies for On-Surface Synthesis. Chem. Rev. 2019, 119, 4717–4776. 10.1021/acs.chemrev.8b00601. 30875199 PMC6477809

[ref36] Grill L.; Hecht S. Covalent On-Surface Polymerization. Nat. Chem. 2020, 12, 115–130. 10.1038/s41557-019-0392-9. 31996811

[ref37] Li X.; Ge H.; Xue R.; Wu M.; Chi L. Anchoring and Reacting On-Surface to Achieve Programmability. JACS Au 2022, 2, 58–65. 10.1021/jacsau.1c00397. 35098221 PMC8790738

[ref38] Wang T.; Fan Q.; Zhu J. Steering On-Surface Reactions by Kinetic and Thermodynamic Strategies. J. Phys. Chem. Lett. 2023, 14, 2251–2262. 10.1021/acs.jpclett.3c00001. 36821589

[ref39] Gao H.; Wagner H.; Zhong D.; Franke J.; Studer A.; Fuchs H. Glaser Coupling at Metal Surfaces. Angew. Chem., Int. Ed. 2013, 52, 4024–4028. 10.1002/anie.201208597. 23424176

[ref40] Liu J.; Chen Q.; Xiao L.; Shang J.; Zhou X.; Zhang Y.; Wang Y.; Shao X.; Li J.; Chen W.; Xu G. Q.; Tang H.; Zhao D.; Wu K. Lattice-Directed Formation of Covalent and Organometallic Molecular Wires by Terminal Alkynes on Ag Surfaces. ACS Nano 2015, 9, 6305–6314. 10.1021/acsnano.5b01803. 25990647

[ref41] Klappenberger F.; Zhang Y.-Q.; Björk J.; Klyatskaya S.; Ruben M.; Barth J. V. On-Surface Synthesis of Carbon-Based Scaffolds and Nanomaterials Using Terminal Alkynes. Acc. Chem. Res. 2015, 48, 2140–2150. 10.1021/acs.accounts.5b00174. 26156663

[ref42] Sun Q.; Cai L.; Ma H.; Yuan C.; Xu W. Dehalogenative Homocoupling of Terminal Alkynyl Bromides on Au(111): Incorporation of Acetylenic Scaffolding into Surface Nanostructures. ACS Nano 2016, 10, 7023–7030. 10.1021/acsnano.6b03048. 27326451

[ref43] Wang T.; Huang J.; Lv H.; Fan Q.; Feng L.; Tao Z.; Ju H.; Wu X.; Tait S. L.; Zhu J. Kinetic Strategies for the Formation of Graphyne Nanowires via Sonogashira Coupling on Ag(111). J. Am. Chem. Soc. 2018, 140, 13421–13428. 10.1021/jacs.8b08477. 30240562

[ref44] Wang T.; Lv H.; Huang J.; Shan H.; Feng L.; Mao Y.; Wang J.; Zhang W.; Han D.; Xu Q.; Du P.; Zhao A.; Wu X.; Tait S. L.; Zhu J. Reaction Selectivity of Homochiral versus Heterochiral Intermolecular Reactions of Prochiral Terminal Alkynes on Surfaces. Nat. Commun. 2019, 10, 4122 10.1038/s41467-019-12102-y. 31511503 PMC6739358

[ref45] Albrecht F.; Rey D.; Fatayer S.; Schulz F.; Pérez D.; Peña D.; Gross L. Intramolecular Coupling of Terminal Alkynes by Atom Manipulation. Angew. Chem., Int. Ed. 2020, 59, 22989–22993. 10.1002/anie.202009200. PMC7756451 32845044

[ref46] Shu C.-H.; He Y.; Zhang R.-X.; Chen J.-L.; Wang A.; Liu P.-N. Atomic-Scale Visualization of Stepwise Growth Mechanism of Metal-Alkynyl Networks on Surfaces. J. Am. Chem. Soc. 2020, 142, 16579–16586. 10.1021/jacs.0c04311. 32900189

[ref47] Lawrence J.; Mohammed M. S. G.; Rey D.; Aguilar-Galindo F.; Berdonces-Layunta A.; Peña D.; de Oteyza D. G. Reassessing Alkyne Coupling Reactions While Studying the Electronic Properties of Diverse Pyrene Linkages at Surfaces. ACS Nano 2021, 15, 4937–4946. 10.1021/acsnano.0c09756. 33630588 PMC7992190

[ref48] Yu X.; Sun Q.; Liu M.; Du W.; Liu Y.; Cai L.; Zha Z.; Pan J.; Kang F.; Gao W.; Yang D.; Qiu X.; Xu W. Lattice-Directed Selective Synthesis of Acetylenic and Diacetylenic Organometallic Polyynes. Chem. Mater. 2022, 34, 1770–1777. 10.1021/acs.chemmater.1c04015.

[ref49] Zhang R.; Lyu G.; Li D. Y.; Liu P. N.; Lin N. Template-Controlled Sonogashira Cross-Coupling Reactions on a Au(111) Surface. Chem. Commun. 2017, 53, 1731–1734. 10.1039/C6CC10091K. 28106195

[ref50] Kang F.; Xu W. On-Surface Synthesis of One-Dimensional Carbon-Based Nanostructures via C–X and C–H Activation Reactions. ChemPhysChem 2019, 20, 2251–2261. 10.1002/cphc.201900266. 31081259

[ref51] Zhang H.; Song C.; Lyu Y.; Cheng P.; Chen L.; Zhang C.; Meng S.; Wu K.; Zhang Y.-Q. Radical-Promoted Room-Temperature Terminal Alkyne Activation on Au(111). Surf. Sci. 2023, 727, 122180 10.1016/j.susc.2022.122180.

[ref52] Kawai S.; Krejčí O.; Foster A. S.; Pawlak R.; Xu F.; Peng L.; Orita A.; Meyer E. Diacetylene Linked Anthracene Oligomers Synthesized by One-Shot Homocoupling of Trimethylsilyl on Cu(111). ACS Nano 2018, 12, 8791–8797. 10.1021/acsnano.8b05116. 30086235

[ref53] Zhang L.; Zhang Y.; Chen Z.; Lin T.; Paszkiewicz M.; Hellwig R.; Huang T.; Ruben M.; Barth J. V.; Klappenberger F. On-Surface Activation of Trimethylsilyl-Terminated Alkynes on Coinage Metal Surfaces. ChemPhysChem 2019, 20, 2382–2393. 10.1002/cphc.201900249. 31120616

[ref54] Jiménez-Martín A.; Villalobos F.; Mallada B.; Edalatmanesh S.; Matěj A.; Cuerva J. M.; Jelínek P.; Campaña A. G.; de la Torre B. On-Surface Synthesis of Non-Benzenoid Conjugated Polymers by Selective Atomic Rearrangement of Ethynylarenes. Chem. Sci. 2023, 14, 1403–1412. 10.1039/D2SC04722E. 36794197 PMC9906656

[ref55] Kong H.; Viergutz L.; Liu L.; Sandvoß A.; Peng X.; Klaasen H.; Fuchs H.; Studer A. Highly Selective On-Surface Reactions of Aryl Propiolic Acids via Decarboxylative Coupling. Adv. Mater. 2023, 35, e2210997 10.1002/adma.202210997. 36740777

[ref56] Cao N.; Yang B.; Riss A.; Rosen J.; Björk J.; Barth J. V. On-Surface Synthesis of Enetriynes. Nat. Commun. 2023, 14, 1255 10.1038/s41467-023-36828-y. 36878914 PMC9988975

[ref57] Wang T.; Lv H.; Fan Q.; Feng L.; Wu X.; Zhu J. Highly Selective Synthesis of cis-Enediynes on a Ag(111) Surface. Angew. Chem., Int. Ed. 2017, 56, 4762–4766. 10.1002/anie.201701142. 28345286

[ref58] Li Q.; Gao J.; Li Y.; Fuentes-Cabrera M.; Liu M.; Qiu X.; Lin H.; Chi L.; Pan M. Self-Assembly Directed One-Step Synthesis of [4]Radialene on Cu(100) Surfaces. Nat. Commun. 2018, 9, 3113 10.1038/s41467-018-05472-2. 30082699 PMC6078953

[ref59] Wang T.; Pan Y.; Zhang W.; Lawrence J.; Mohammed M. S. G.; Huang J.; Feng L.; Berdonces-Layunta A.; Han D.; Xu Q.; Wu X.; Tait S. L.; de Oteyza D. G.; Zhu J. On-Surface Synthesis of a Five-Membered Carbon Ring from a Terminal Alkynyl Bromide: A [4 + 1] Annulation. J. Phys. Chem. Lett. 2020, 11, 5902–5907. 10.1021/acs.jpclett.0c01483. 32633516

[ref60] Sun Q.; Zhang C.; Li Z.; Kong H.; Tan Q.; Hu A.; Xu W. On-Surface Formation of One-Dimensional Polyphenylene through Bergman Cyclization. J. Am. Chem. Soc. 2013, 135, 8448–8451. 10.1021/ja404039t. 23706147

[ref62] de Oteyza D. G.; Pérez Paz A.; Chen Y.-C.; Pedramrazi Z.; Riss A.; Wickenburg S.; Tsai H.-Z.; Fischer F. R.; Crommie M. F.; Rubio A. Noncovalent Dimerization after Enediyne Cyclization on Au(111). J. Am. Chem. Soc. 2016, 138, 10963–10967. 10.1021/jacs.6b05203. 27490459

[ref63] Schuler B.; Fatayer S.; Mohn F.; Moll N.; Pavliček N.; Meyer G.; Peña D.; Gross L. Reversible Bergman Cyclization by Atomic Manipulation. Nat. Chem. 2016, 8, 220–224. 10.1038/nchem.2438. 26892552

[ref635] Meng X.; Liu L.; Garcia F.; Alvarez B.; Perez D.; Gao H.-Y.; Pena D.; Fuchs H. Effect of Central pi System in Silylated-Tetraynes on sigma Bond Metathesis on Surfaces. J. Phys. Chem. C 2018, 122, 6230–6235. 10.1021/acs.jpcc.8b00519.

[ref64] de Oteyza D. G.; Gorman P.; Chen Y.-C.; Wickenburg S.; Riss A.; Mowbray D. J.; Etkin G.; Pedramrazi Z.; Tsai H.-Z.; Rubio A.; Crommie M. F.; Fischer F. R. Direct Imaging of Covalent Bond Structure in Single-Molecule Chemical Reactions. Science 2013, 340, 1434–1437. 10.1126/science.1238187. 23722428

[ref65] Riss A.; Paz A.; Wickenburg S.; Tsai H.-Z.; de Oteyza D. G.; Bradley A. J.; Ugeda M. M.; Gorman P.; Jung H.; Crommie M. F.; Rubio A.; Fischer F. R. Imaging Single-Molecule Reaction Intermediates Stabilized by Surface Dissipation and Entropy. Nat. Chem. 2016, 8, 678–683. 10.1038/nchem.2506. 27325094

[ref66] Prall M.; Wittkopp A.; Schreiner P. R. Can Fulvenes Form from Enediynes? A Systematic High-Level Computational Study on Parent and Benzannelated Enediyne and Enyne–Allene Cyclizations. J. Phys. Chem. A 2001, 105, 9265–9274. 10.1021/jp0028002.

[ref67] Vavilala C.; Byrne N.; Kraml C. M.; Ho D. M.; Pascal R. A. Thermal C1–C5 Diradical Cyclization of Enediynes. J. Am. Chem. Soc. 2008, 130, 13549–13551. 10.1021/ja803413f. 18798628

[ref61] Riss A.; Wickenburg S.; Gorman P.; Tan L. Z.; Tsai H.-Z.; de Oteyza D. G. de; Chen Y.-C.; Bradley A. J.; Ugeda M. M.; Etkin G.; Louie S. G.; Fischer F. R.; Crommie M. F. Local Electronic and Chemical Structure of Oligo-Acetylene Derivatives Formed Through Radical Cyclizations at a Surface. Nano Lett. 2014, 14, 2251–2255. 10.1021/nl403791q. 24387223 PMC4022646

[ref68] Díaz Arado O.; Mönig H.; Wagner H.; Franke J.-H.; Langewisch G.; Held P. A.; Studer A.; Fuchs H. On-Surface Azide–Alkyne Cycloaddition on Au(111). ACS Nano 2013, 7, 8509–8515. 10.1021/nn4022789. 24047459

[ref69] Bebensee F.; Bombis C.; Vadapoo S.-R.; Cramer J. R.; Besenbacher F.; Gothelf K. V.; Linderoth T. R. On-Surface Azide–Alkyne Cycloaddition on Cu(111): Does It “Click” in Ultrahigh Vacuum?. J. Am. Chem. Soc. 2013, 135, 2136–2139. 10.1021/ja312303a. 23360358

[ref70] Stolz S.; Bauer M.; Pignedoli C. A.; Krane N.; Bommert M.; Turco E.; Bassi N.; Kinikar A.; Merino-Dìez N.; Hany R.; Brune H.; Gröning O.; Widmer R. Asymmetric Azide-Alkyne Huisgen Cycloaddition on Chiral Metal Surfaces. Commun. Chem. 2021, 4, 51 10.1038/s42004-021-00488-0. 36697612 PMC9814088

[ref72] Liu J.; Ruffieux P.; Feng X.; Müllen K.; Fasel R. Cyclotrimerization of Arylalkynes on Au(111). Chem. Commun. 2014, 50, 11200–11203. 10.1039/C4CC02859G. 25110877

[ref73] See Supporting Information for details.

[ref74] Kinikar A.; Giovannantonio M. D.; Urgel J. I.; Eimre K.; Qiu Z.; Gu Y.; Jin E.; Narita A.; Wang X.-Y.; Müllen K.; Ruffieux P.; Pignedoli C. A.; Fasel R. On-Surface Polyarylene Synthesis by Cycloaromatization of Isopropyl Substituents. Nat. Synth. 2022, 1, 289–296. 10.1038/s44160-022-00032-5.

[ref75] aWu J.; Kroll P.; Dias H. V. R. Gold(I) Chloride Coordinated 3-Hexyne. Inorg. Chem. 2009, 48, 423–425. 10.1021/ic8020854. 19072612

[ref76] Gold-to-ligand bond lengths are 1.224 Å for (η^2^-Et-C≡C-Et)AuCl,^75a^ 1.220 Å for [(tBu_3_P)Au^+^(η^2^-tBu-C≡C-CH_3_)][SbF^6–^],^75b^ 1.233 Å for [N{(C_3_F_7_)C(Dipp)N}_2_]Au(EtC≡CEt),^75c^ and 1.220 Å for (cyclododecyne)AuCl.^75d^

[ref77] Alkyne bending angles are 163.0 and 166.9° for (η^2^- Et-C≡C-Et)AuCl,^75a^ 165.6 and 168.1° for [(*t*Bu3P)Au^+^(η^2^-*t*Bu-C≡C-CH_3_)][SbF^6–^],^75b^ 155.0 and 155.7° for [N{(C_3_F_7_)C(Dipp)N}_2_]Au(EtC≡CEt),^75c^ and 165.2 and 165.7° for (cyclododecyne)AuCl.^75d^

[ref78] Ouellette R. J.; Rawn J. D. Organic Chemistry: Structure, Mechanism, Synthesis, 2nd Ed.; Academic Press, 2018.

[ref79] Pranckevicius C.; Liu L. L.; Bertrand G.; Stephan D. W. Synthesis of a Carbodicyclopropenylidene: A Carbodicarbene Based Solely on Carbon. Angew. Chem., Int. Ed. 2016, 55, 5536–5540. 10.1002/anie.201600765. 27028936

[ref80] Fürstner A.; Alcarazo M.; Goddard R.; Lehmann C. W. Coordination Chemistry of Ene-1,1-diamines and a Prototype “Carbodicarbene”. Angew. Chem. Int. Ed 2008, 47, 3210–3214. 10.1002/anie.200705798. 18348113

[ref81] aRupf S.; Pröhm P.; Malischewski M. The [2 + 2] Cycloaddition Product of Perhalogenated Cyclopentadienyl Cations: Structural Characterization of Salts of the [C_10_Cl_10_]^2+^ and [C_10_Br_10_]^2+^ Dications. Chem. Commun. 2020, 56, 9834–9837. 10.1039/D0CC04226A. 32716428

[ref82] Vajda E.; Tremmel J.; Rozsondai B.; Hargittai I.; Maltsev A. K.; Kagramanov N. D.; Nefedov O. M. Molecular Structure of Allyl Radical from Electron Diffraction. J. Am. Chem. Soc. 1986, 108, 4352–4353. 10.1021/ja00275a020.

[ref83] Gomes G.; Dos P.; Alabugin I. V. Drawing Catalytic Power from Charge Separation: Stereoelectronic and Zwitterionic Assistance in the Au(I)-Catalyzed Bergman Cyclization. J. Am. Chem. Soc. 2017, 139, 3406–3416. 10.1021/jacs.6b11054. 28187258

[ref84] Hashmi A. S. K.; Braun I.; Rudolph M.; Rominger F. The Role of Gold Acetylides as a Selectivity Trigger and the Importance of Gem-Diaurated Species in the Gold-Catalyzed Hydroarylating-Aromatization of Arene-Diynes. Organometallics 2012, 31, 644–661. 10.1021/om200946m.

[ref85] Naoe S.; Suzuki Y.; Hirano K.; Inaba Y.; Oishi S.; Fujii N.; Ohno H. Gold(I)-Catalyzed Regioselective Inter-/Intramolecular Addition Cascade of Di- and Triynes for Direct Construction of Substituted Naphthalenes. J. Org. Chem. 2012, 77, 4907–4916. 10.1021/jo300771f. 22568806

[ref86] Alabugin I. V.; Gonzalez-Rodriguez E. Alkyne Origami: Folding Oligoalkynes into Polyaromatics. Acc. Chem. Res. 2018, 51, 1206–1219. 10.1021/acs.accounts.8b00026. 29676896

[ref87] Guerra C. F.; Handgraaf J.; Baerends E. J.; Bickelhaupt F. M. Voronoi Deformation Density (VDD) Charges: Assessment of the Mulliken, Bader, Hirshfeld, Weinhold, and VDD Methods for Charge Analysis. J. Comput. Chem. 2004, 25, 189–210. 10.1002/jcc.10351. 14648618

[ref88] Bronner C.; Marangoni T.; Rizzo D. J.; Durr R. A.; Jo̷rgensen J. H.; Fischer F. R.; Crommie M. F. Iodine versus Bromine Functionalization for Bottom-Up Graphene Nanoribbon Growth: Role of Diffusion. J. Phys. Chem. C 2017, 121, 18490–18495. 10.1021/acs.jpcc.7b02896.

[ref89] Periasamy M.; Karunakar G. V.; Bharathi P. Synthesis of Enynones from Alkynes, Alkynyl Ketones and Aromatic Aldehydes Using the TiCl_4_/Et_3_N Reagent System. J. Chem. Res. 2006, 2006, 566–568. 10.3184/030823406778521446s.

