# Supplementary material for: Dramatic Acceleration of the Hopf Cyclization on Gold(111): From Enediynes to Peri-Fused Diindenochrysene Graphene Nanoribbons
Source: J Am Chem Soc. 2024 Jan 16;146(4):2474–83. doi: 10.1021/jacs.3c10144 (PMC10835731; doi:10.1021/jacs.3c10144)
Supplement: Supplementary file 1 — ja3c10144_si_001.pdf [file ja3c10144_si_001.pdf]

Supporting Information for

Dramatic Acceleration of the Hopf Cyclization on Gold(111): From Enediynes to Peri-fused Diindenochrysene Graphene Nanoribbons

Chenxiao Zhao,[1] Dayanni D. Bhagwandin,[2] Wangwei Xu,[1] Pascal Ruffieux,[1] Saeed I. Khan,[2] Carlo A. Pignedoli,*,[1] Roman Fasel*,[1,3] and Yves Rubin*,[2]

[1]Empa—Swiss Federal Laboratories for Materials Science and Technology, Dübendorf, Switzerland
[2]Department of Chemistry and Biochemistry, University of California, Los Angeles, 607 Charles Young Dr. East, Los Angeles, California 90095-1567, United States
[3]University of Bern, Bern, Switzerland

Table of Contents

Section 1: SI Figures Mentioned in the Text S2

Section 2: Experimental Details S15

2.1. Synthesis of (*E*)-1,3,4,6-tetraphenyl-3-hexen-1,5-diyne (**1a**) and (*E*)-3,4-bis(4-iodophenyl)-1,6-diphenyl-3-hexen-1,5-diyne (**1b**). S15

2.1.1 Experimental procedures for the preparation of (*E*)-1,3,4,6-tetraphenyl-3-hexen-1,5-diyne (**1a**). S15

2.1.2. Experimental procedures for the preparation of (*E*)-3,4-bis(4-iodophenyl)-1,6-diphenyl-3-hexen-1,5-diyne (**1b**). S17

2.2. NMR spectra S19

2.3. STM and nc-AFM measurements S25

Section 3: Computational Details S26

3.1. Gas Phase Calculations S26

3.2. On-Surface Calculations S26

3.3. Band Structure Projected Density of States Calculations S27

Section 4: References S29

Section 1. SI Figures Mentioned in the Text.

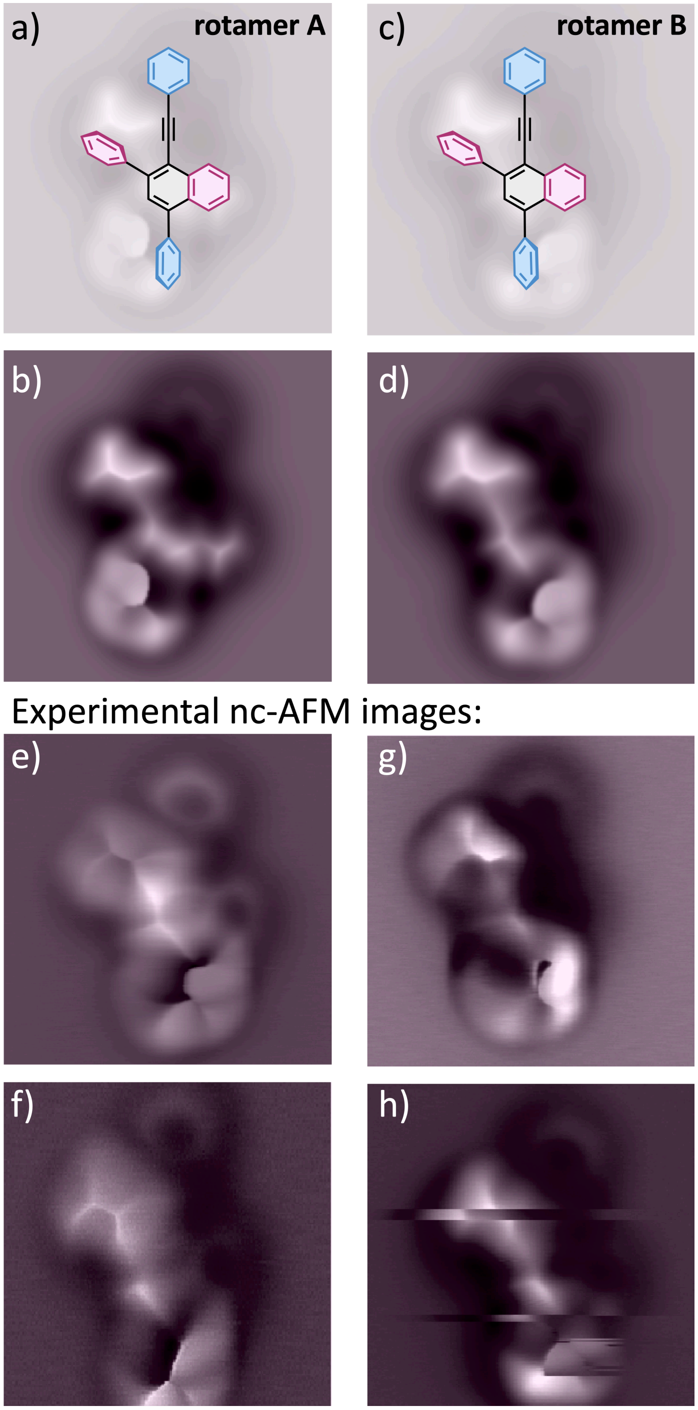

Figure S1. Different rotamers of naphthalene derivative **2** obtained by non-contact AFM at 4 K. a,b) Calculated nc-AFM image of **rotamer A**, with the top edge of the bottom phenyl group oriented to the left. a) Superposition of the structure of naphthalene **2** over the same image as in b) but with an 80% transparency of the nc-AFM image applied for easier visualization. c,d) Calculated nc-AFM image of **rotamer B**, with the top edge of the bottom phenyl group oriented to the right. c) Superposition of the structure of naphthalene **2** over the same image as in d) but with an 80% transparency of the nc-AFM image applied for easier visualization. e-h) Experimental nc-AFM images of derivative **2**, all are in the **rotamer B** configuration.

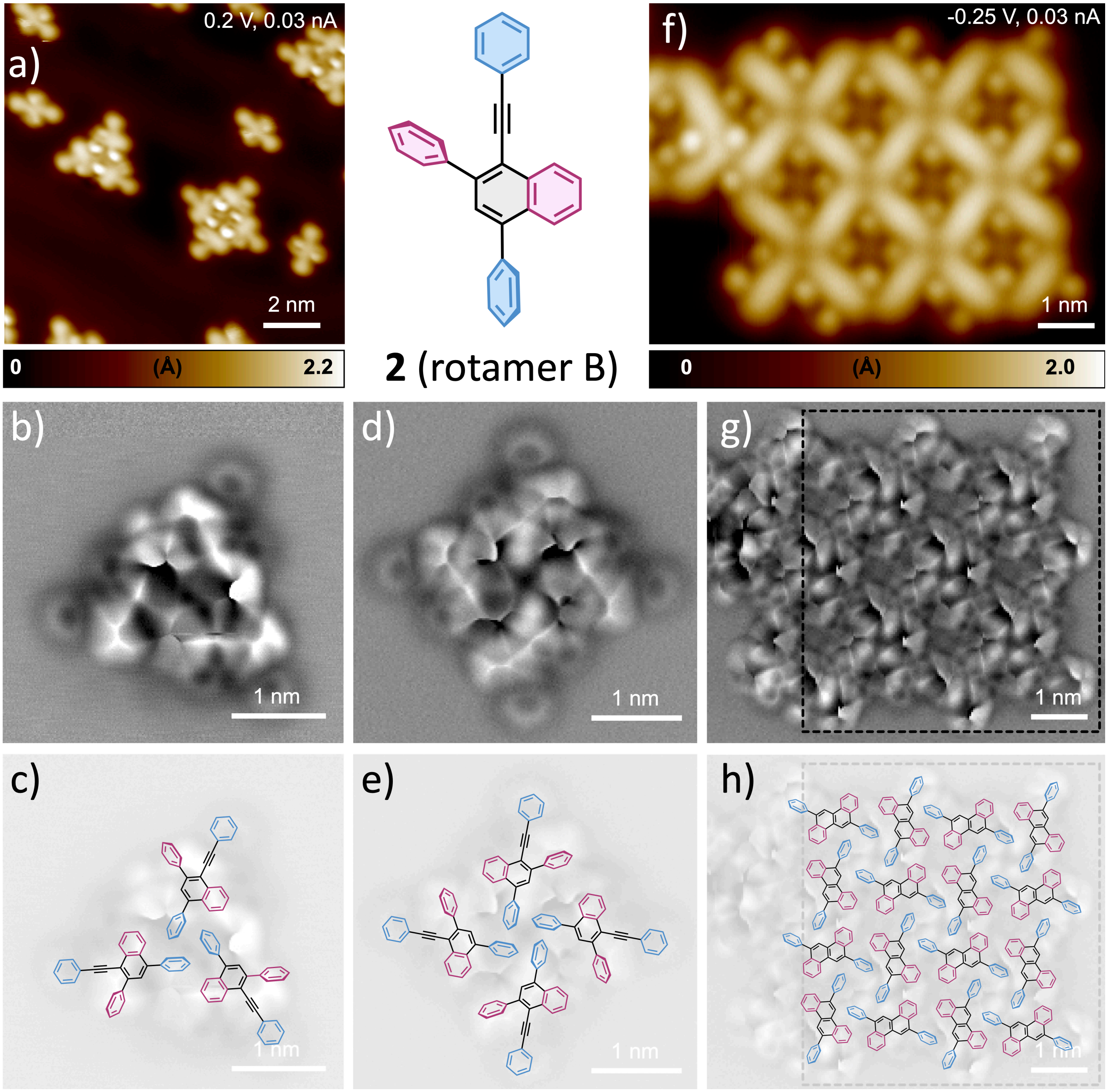

Figure S2. Experimental images of the self-assembled islands of naphthalene derivative **2** on Au(111). a) Overview STM image. b) A trimeric island of **2** imaged by nc-AFM, with c) three superimposed structures of naphthalene **2** over the same image as in b) but with an 80% transparency of the nc-AFM image applied for easier visualization. d) A tetrameric island of **2** imaged by nc-AFM, with e) four superimposed structures of naphthalene **2** over the same image as in d) but with an 80% transparency of the nc-AFM image applied for easier visualization. f) STM image of a large cluster of chrysene derivative **3**. g) The same island of **3**, shown in f), imaged by nc-AFM. h) Superimposed structures of naphthalene **3** over the same image as in g) but with an 80% transparency of the nc-AFM image applied for easier visualization.

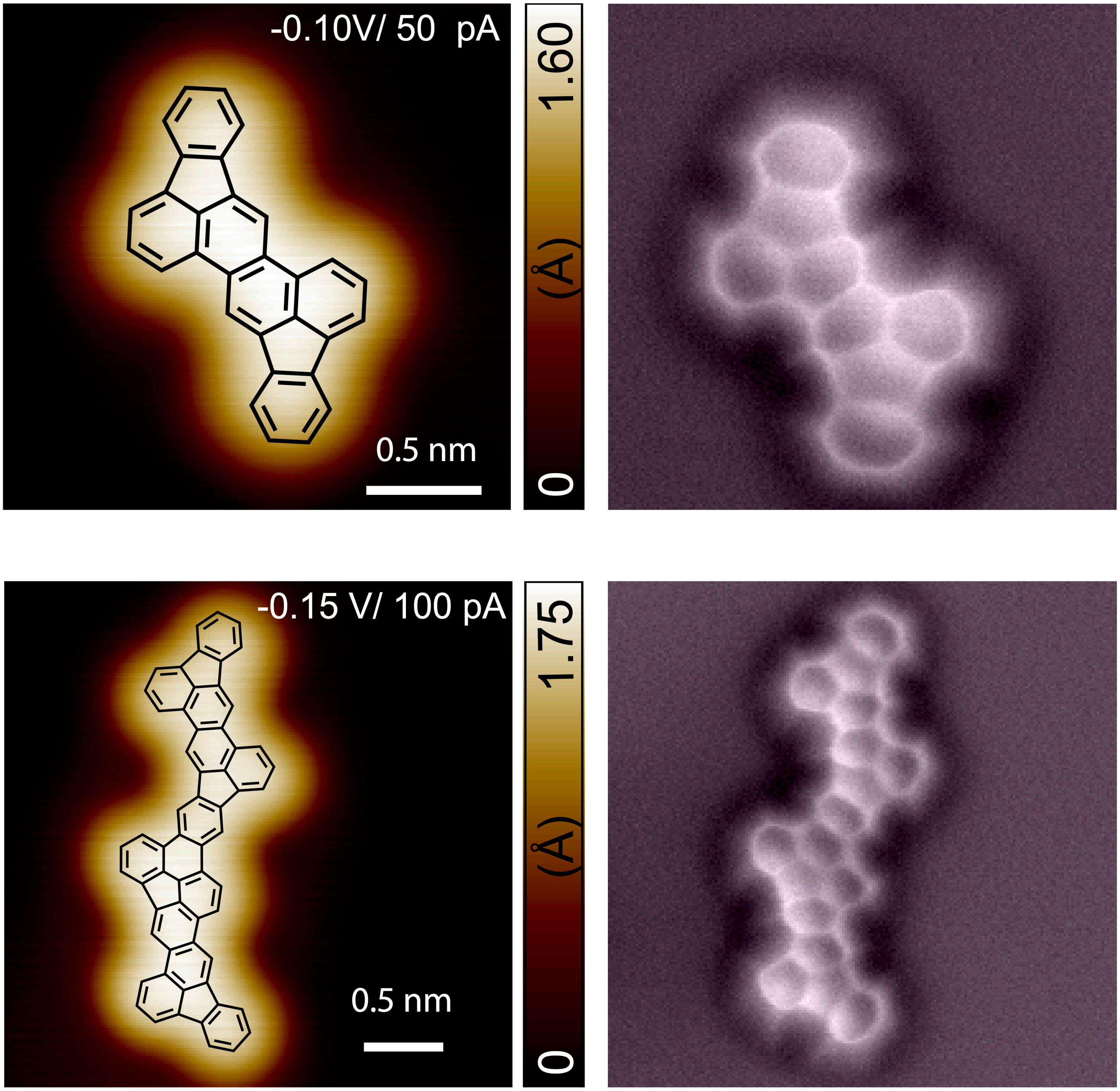

Figure S3. Topographic STM (left) and nc-AFM (right) images of post-Hopf oligomers formed on Au(111) after heating deposited enediyne **1a** at 300 ˚C for 15 minutes. The structures corresponding to the observed oligomers are superposed over the topographic STM images at left.

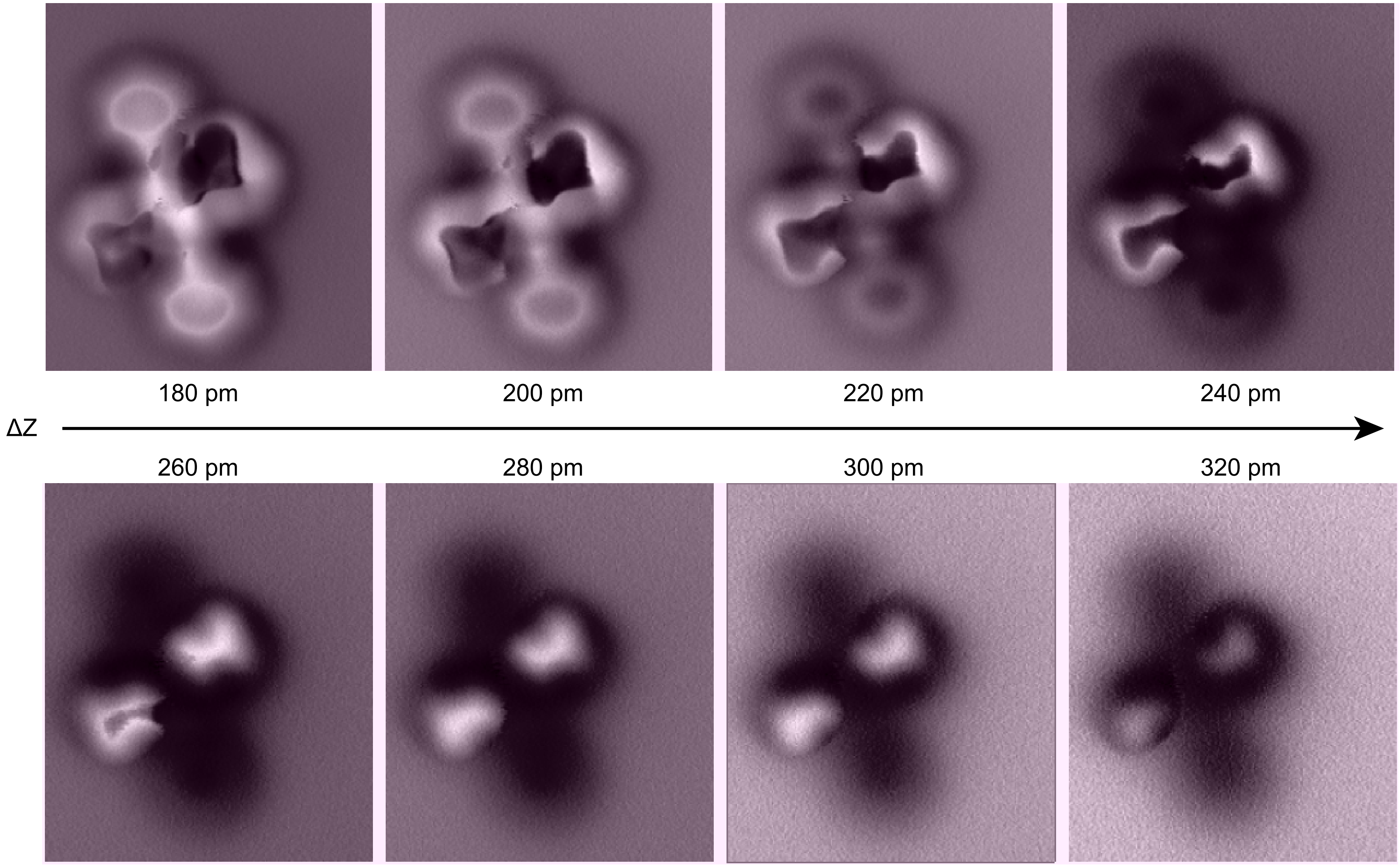

Figure S4. nc-AFM images of **1a** at different tip-molecule distances.

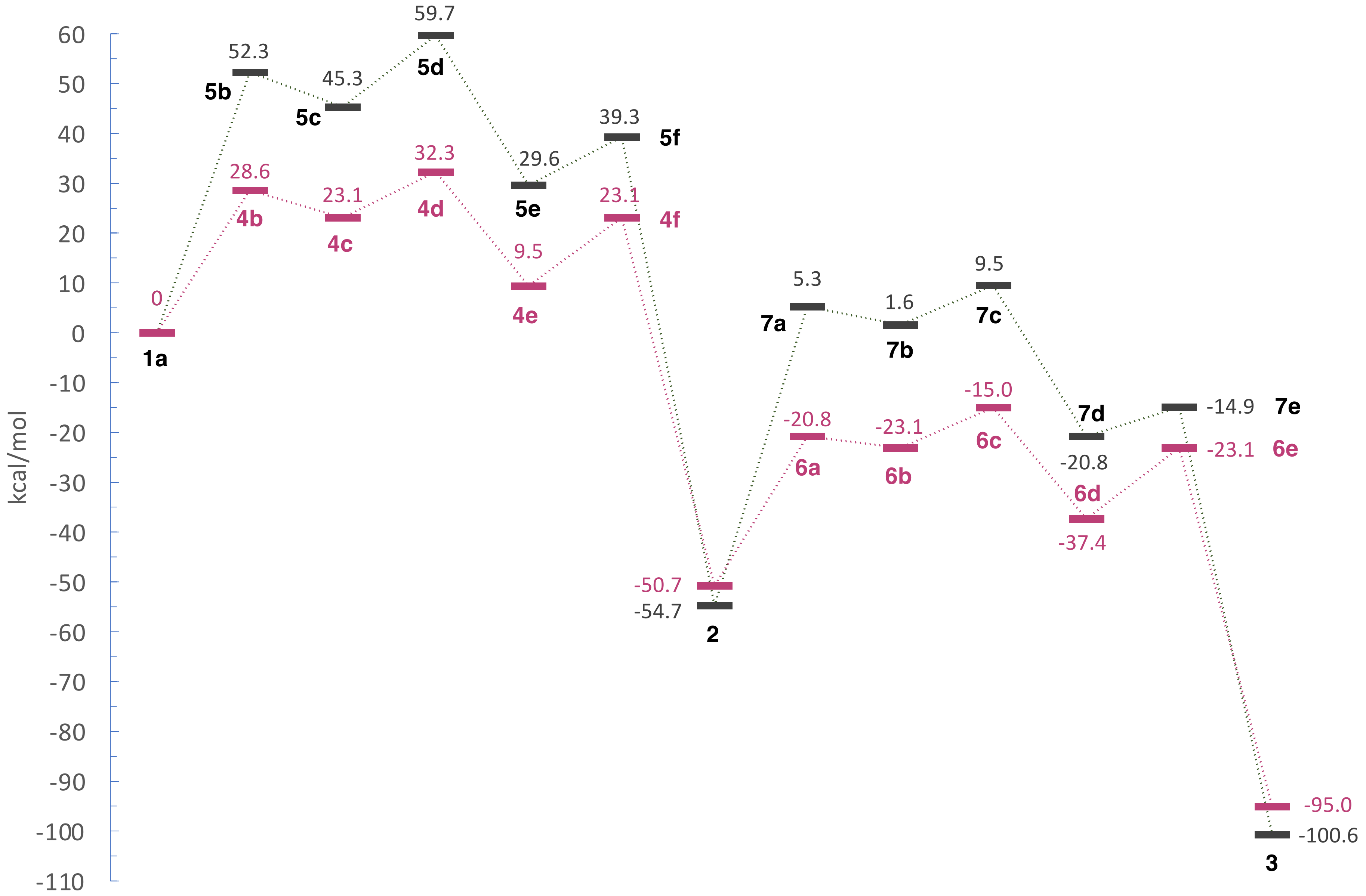

Figure S5. Progression of the Hopf cyclization along the reaction coordinate in the gas phase and on-surface. Values/bars in black are relative free energies in kcal/mol of intermediates in the gas phase, and values/bars in purple are for intermediates on the Au(111) gold slab model (for the structures corresponding to compound numbers, see Fig. 3).

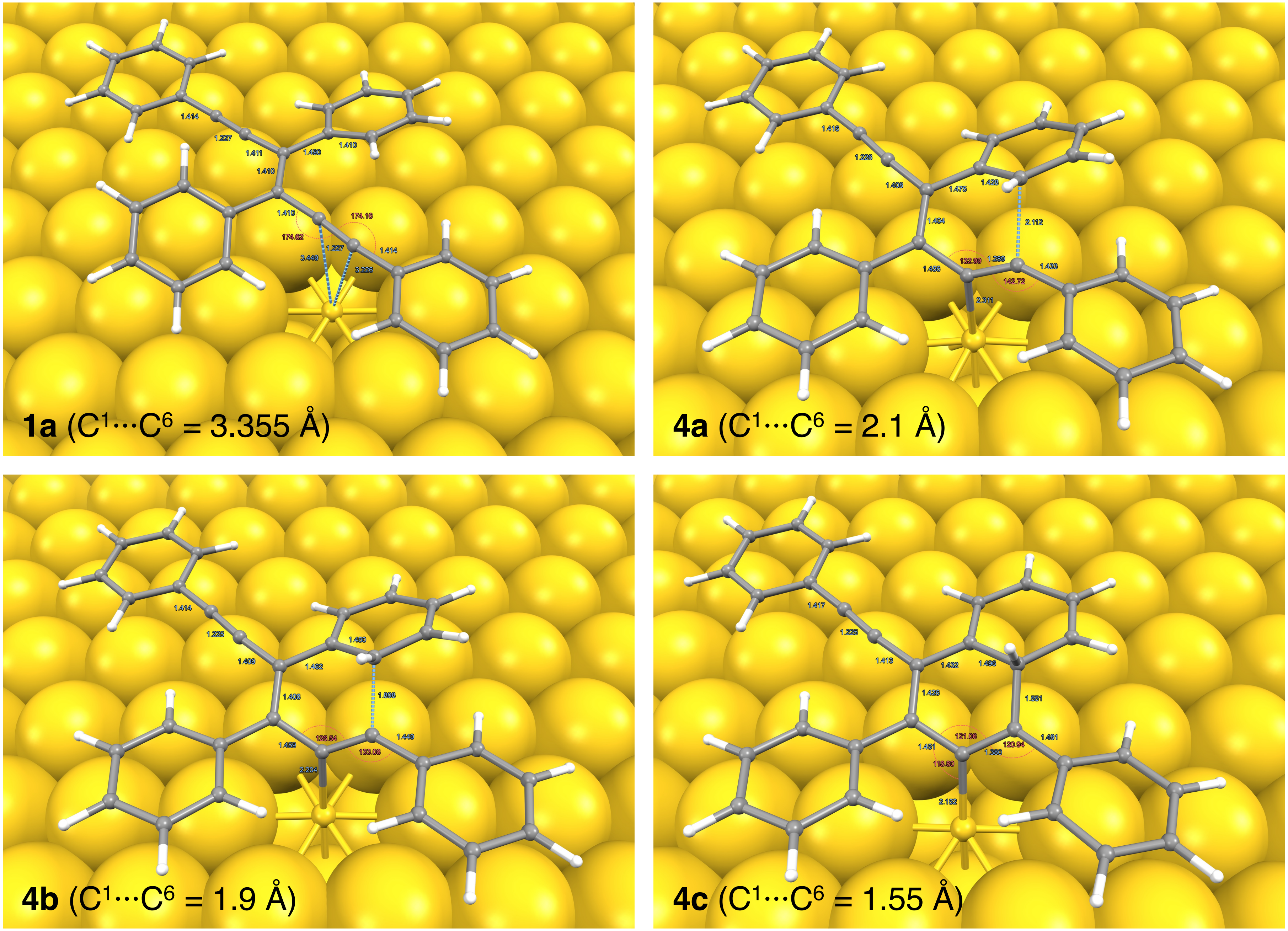

Figure S6A. Structural details for the intermediates obtained during the MEP calculations for the first 6π-electrocyclization step. The $C^1 \cdots C^6$ distance was constrained and decreased in 0.1 Å increments.

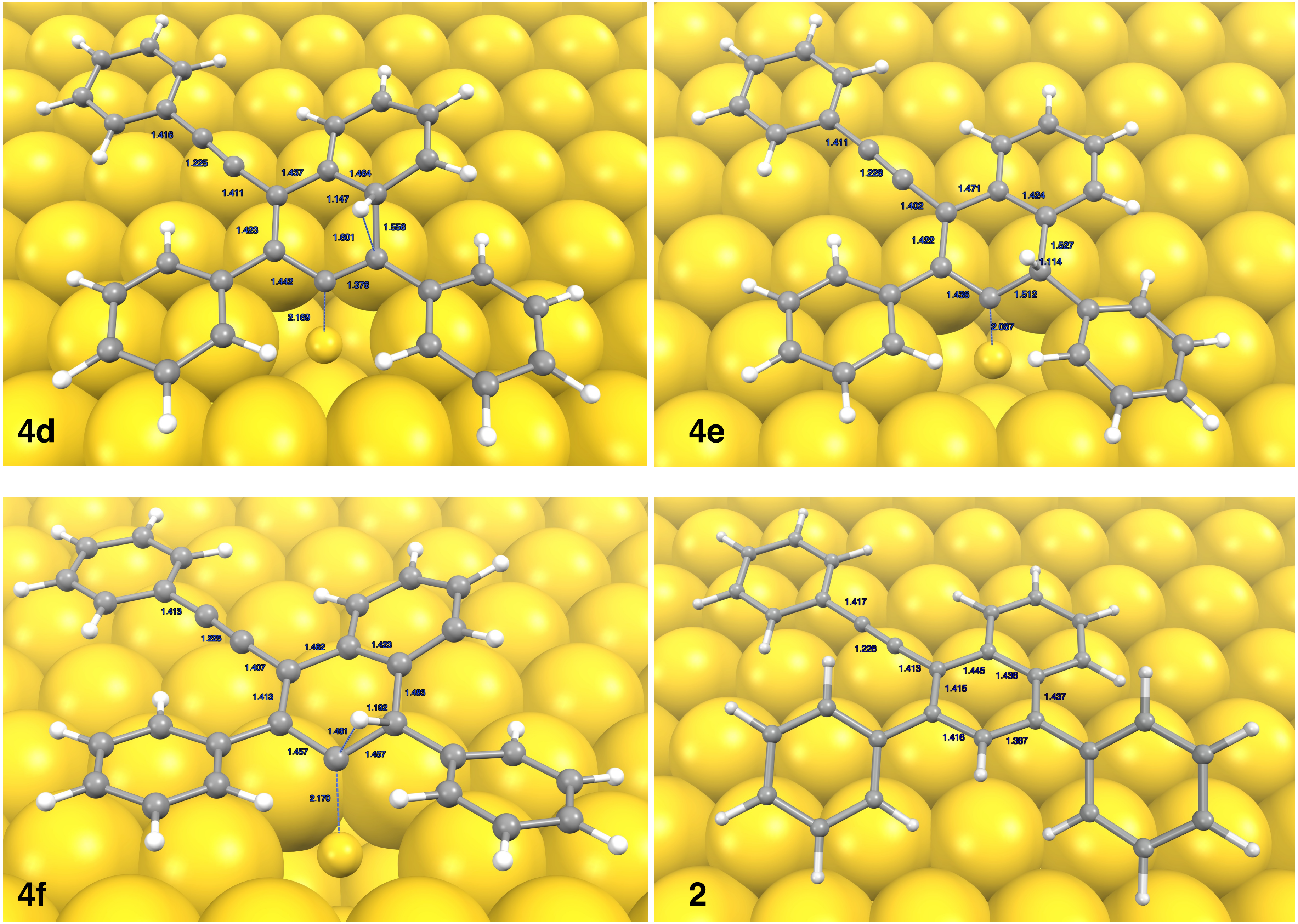

Figure S6B. Structural details for the intermediates obtained during the MEP calculations for the two following 1,2-H shift steps.

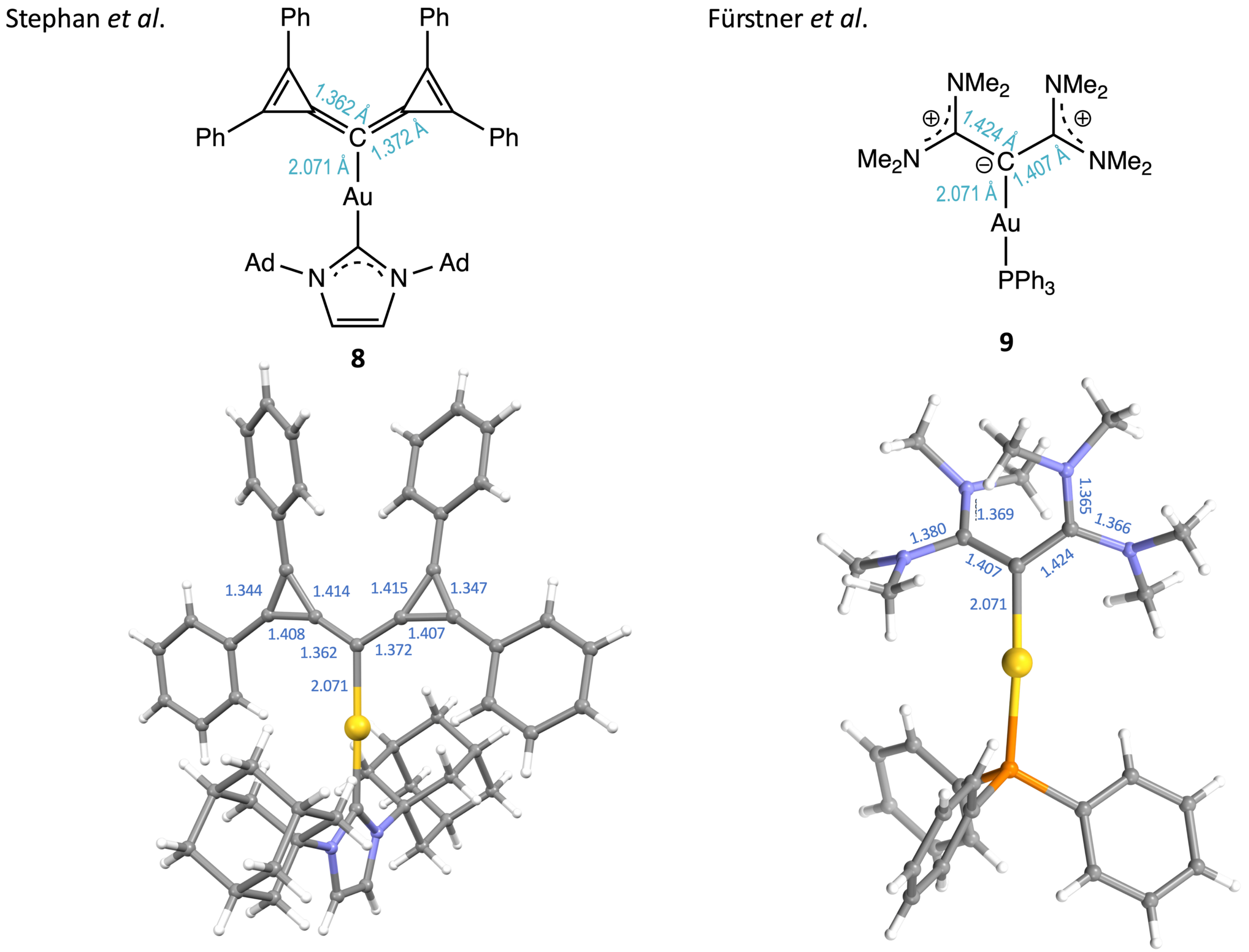

Figure S7. Gold(I) h^1-complexes with allene/carbidodicarbene moieties.[1,2,3,4] CIF crystallographic files were obtained from the CCDC database and plotted with Mercury.[5,6]

Malischewski *et al*.

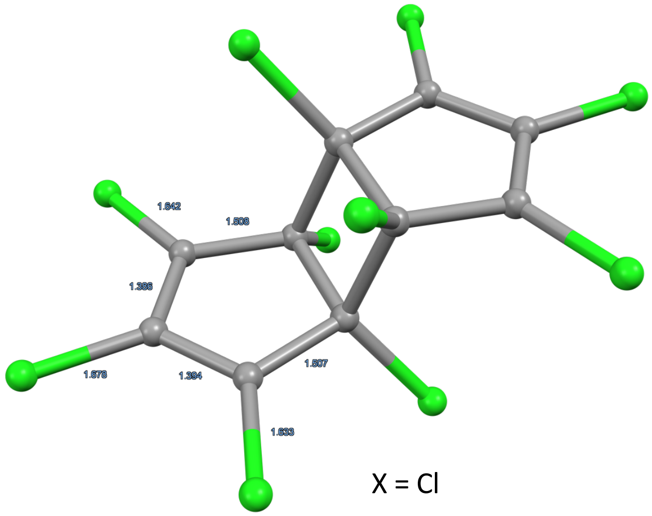

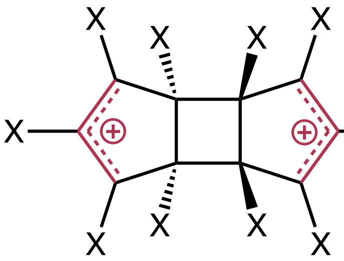

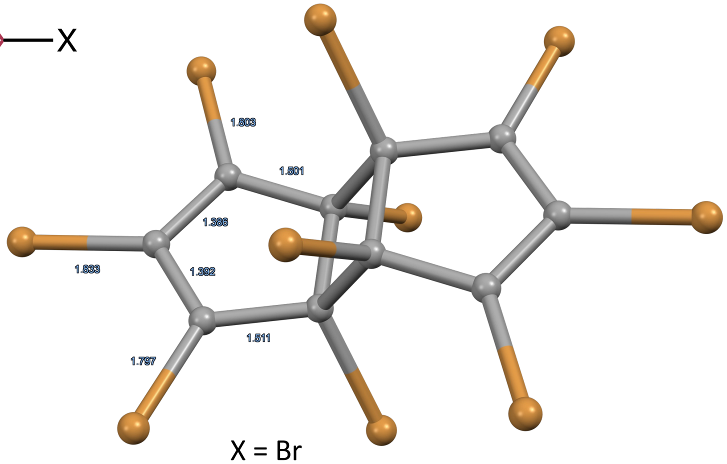

Siegel *et al*.

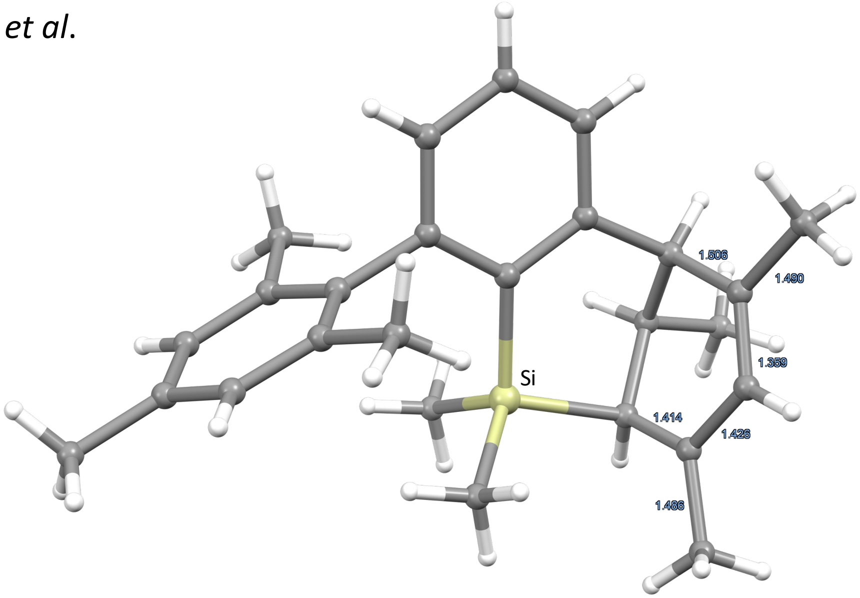

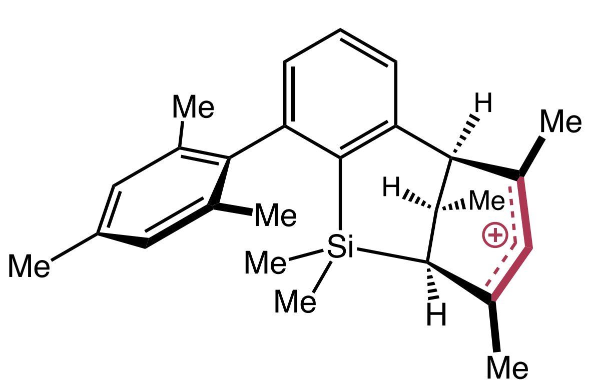

Figure S8. Crystal structures of stable allylic cations.[7] CIF crystallographic files were obtained from the CCDC database.[8,9,10]

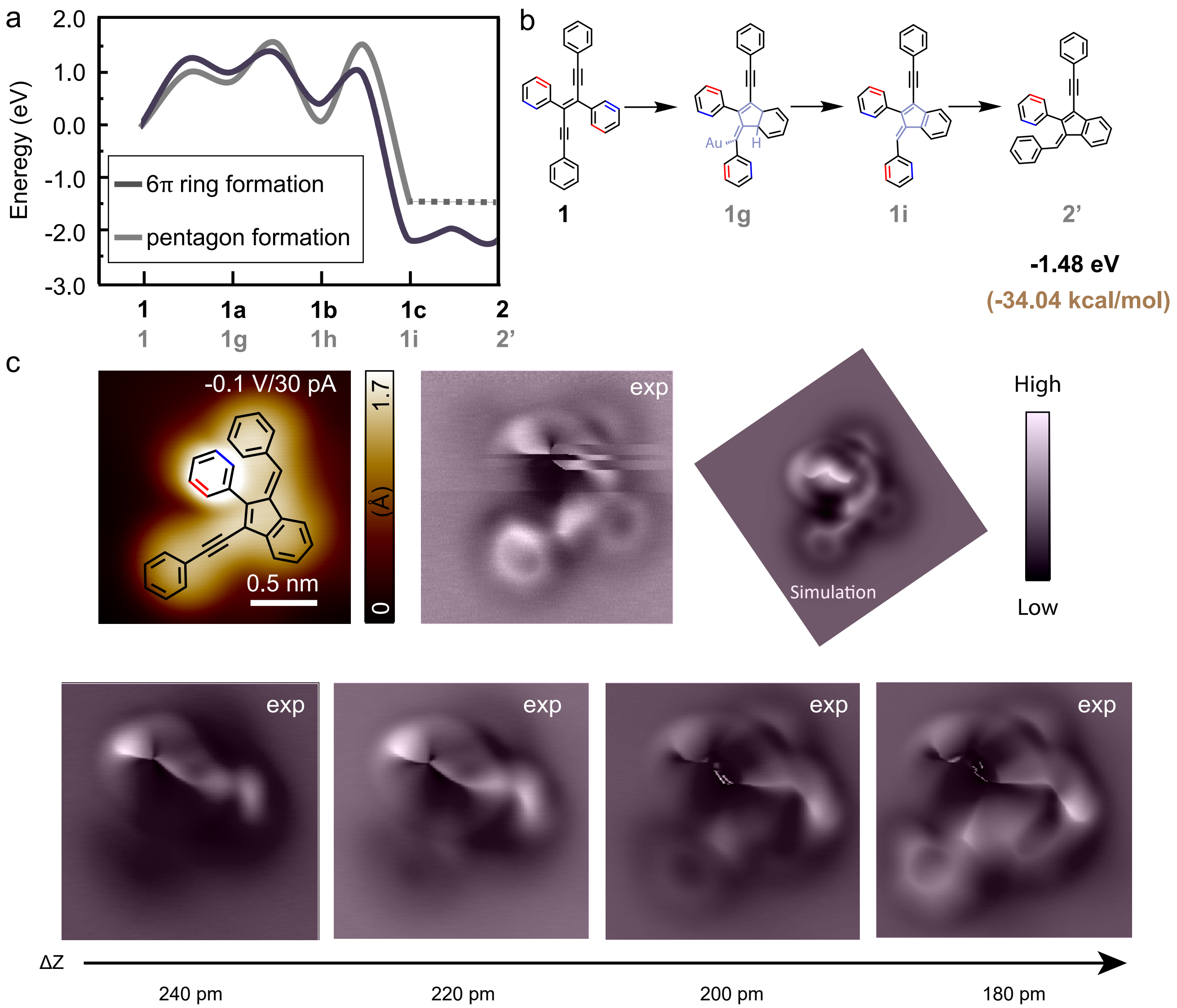

Figure S9. Observation of a rare pentafulvene side-product (**2'**) after heating of enediyne **1a** at 160 ˚C. a) Comparison of the energetic profiles for the calculated 6-endo-dig and 5-exo-dig cyclizations on Au(111). b) Scheme of the overall transformation from **1a** to **2'**, showing the involvement of a gold atom from the surface. The red bonds represent the side of the phenyl group that is oriented up, while the blue bonds are for the sides oriented down. c) STM topographic and nc-AFM images of the pentafulvene side-product. Both simulated and experimental images are shown, with the bottom nc-AFM images displaying images taken at different tip-molecule distances.

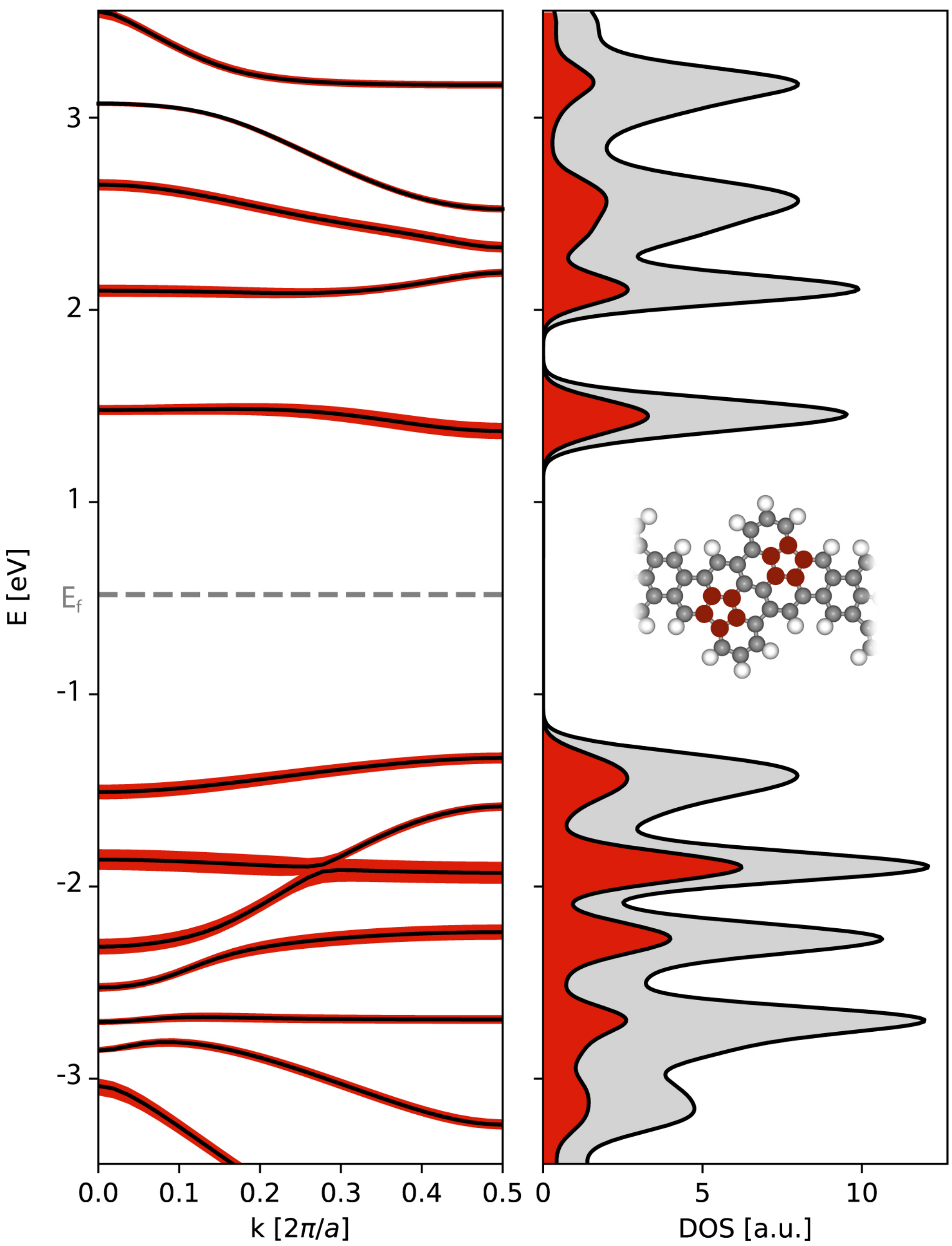

Figure S10. Calculated band structure of graphene nanoribbon **13** (left panel), and projected density of states of the 5-membered ring carbon atoms (right panel). The projected density of states of the pentagon carbon atoms are in red (right panel), while the carbons of the entire nanoribbon are shown in grey. For calculation details, see Section 3.3.

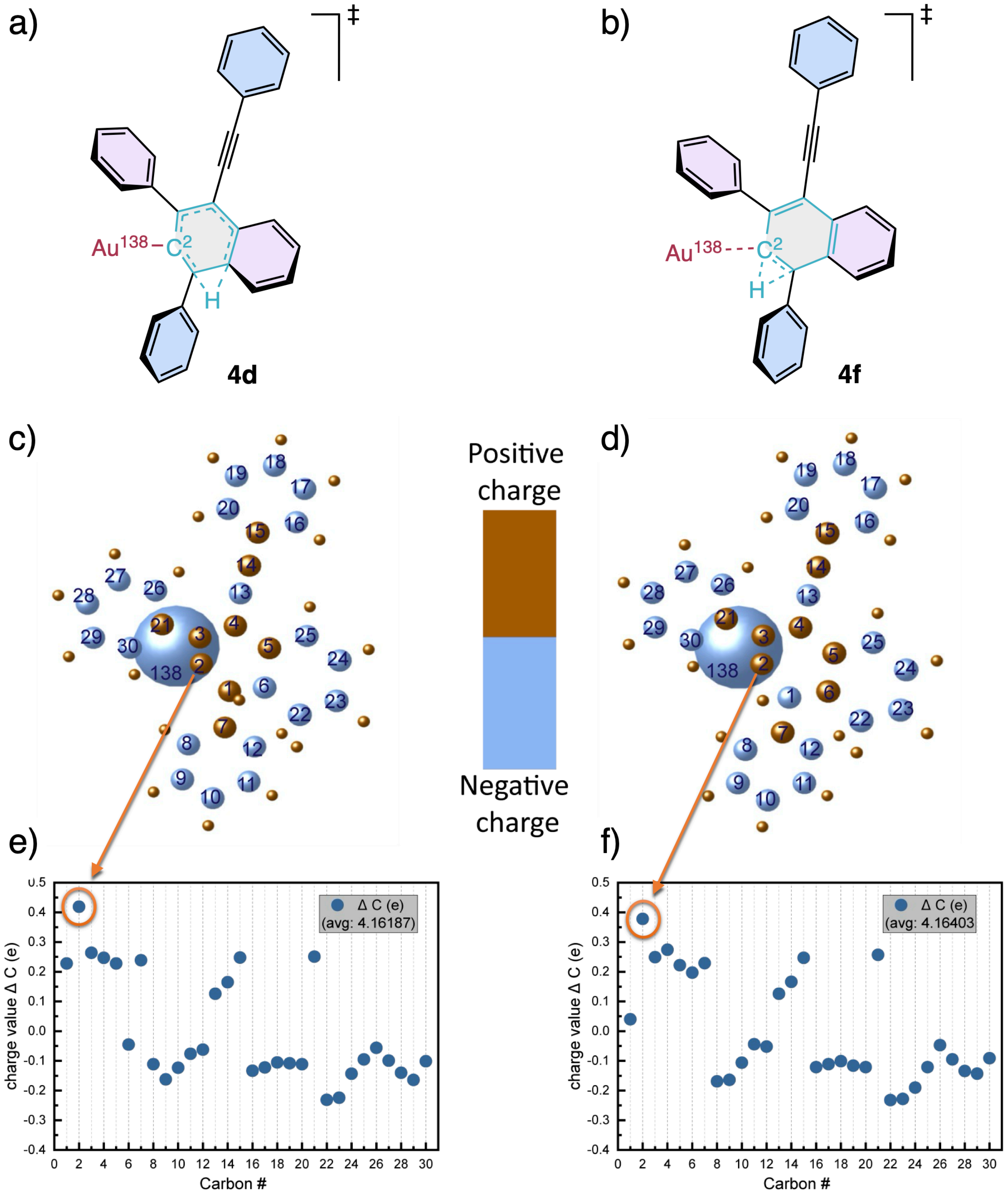

Figure S11. Hirshfeld charge analysis of transition states **4d** and **4f** in the first and second 1,2-H shifts steps. a) and b) Chemical structures of **4d** and **4f**, respectively, positioned to match the orientation of atoms in the Hirshfeld charge plots of c) and d). c), d) Top views of the geometries for transition states **4d** and **4f**, respectively, calculated at the PBE level of theory on a gold slab model. The color-coded spatially resolved charge values for carbons C^1–C^{30} and Au^{138} atoms obtained from a Hirshfeld charge population analysis are shown as spheres whose area is proportional to the charge values. e) and f) Plots of charge deviation values from the average population (avg) for C^1–C^{30} of **4d** and **4f**, respectively.

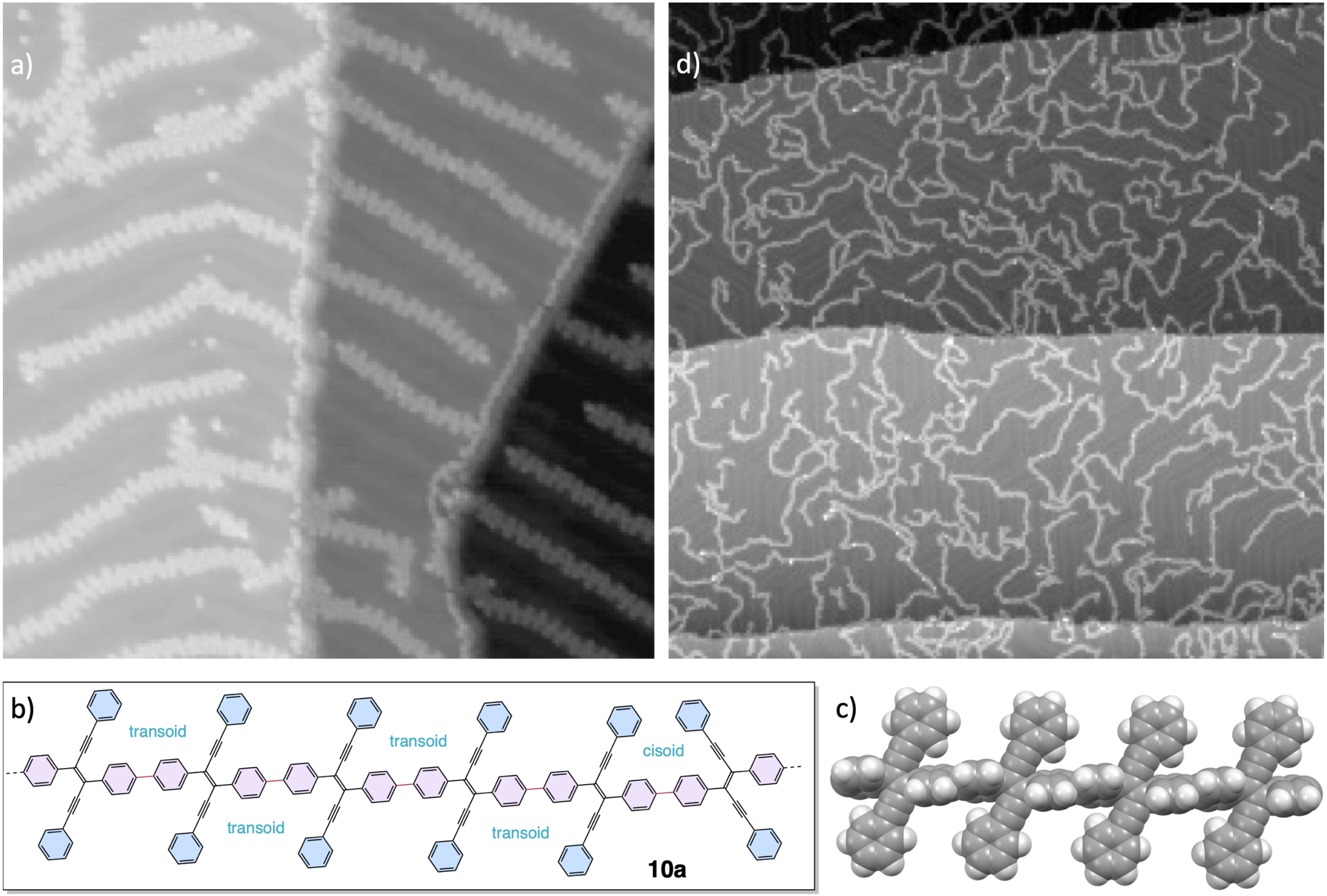

Figure S12. Topographic STM images (a,d) of post-Hopf oligomers formed on Au(111) after heating deposited enediyne **1a** at 300 ˚C for 15 minutes.

Section 2. Experimental details

2.1. Synthesis of (*E*)-1,3,4,6-tetraphenyl-3-hexen-1,5-diyne (1a) and (*E*)-3,4-bis(4-iodophenyl)-1,6-diphenyl-3-hexen-1,5-diyne (1b).

General procedures: Chemical reagents were obtained from commercial sources and used without further purification. Unless stated otherwise, reactions were performed under an argon atmosphere in flame-dried glassware. Solvents such as THF were dried by distillation over sodium/ketyl. Unless otherwise noted, all reactions were performed under argon at 25 ˚C. Column chromatography was performed on Silicycle (Siliflash P60) silica gel 60 (240-400 mesh). Thin layer chromatography utilized pre-coated plates from E. Merck (silica gel 60 PF254, 0.25 mm). NMR spectra were obtained on a Bruker AV500 instrument.

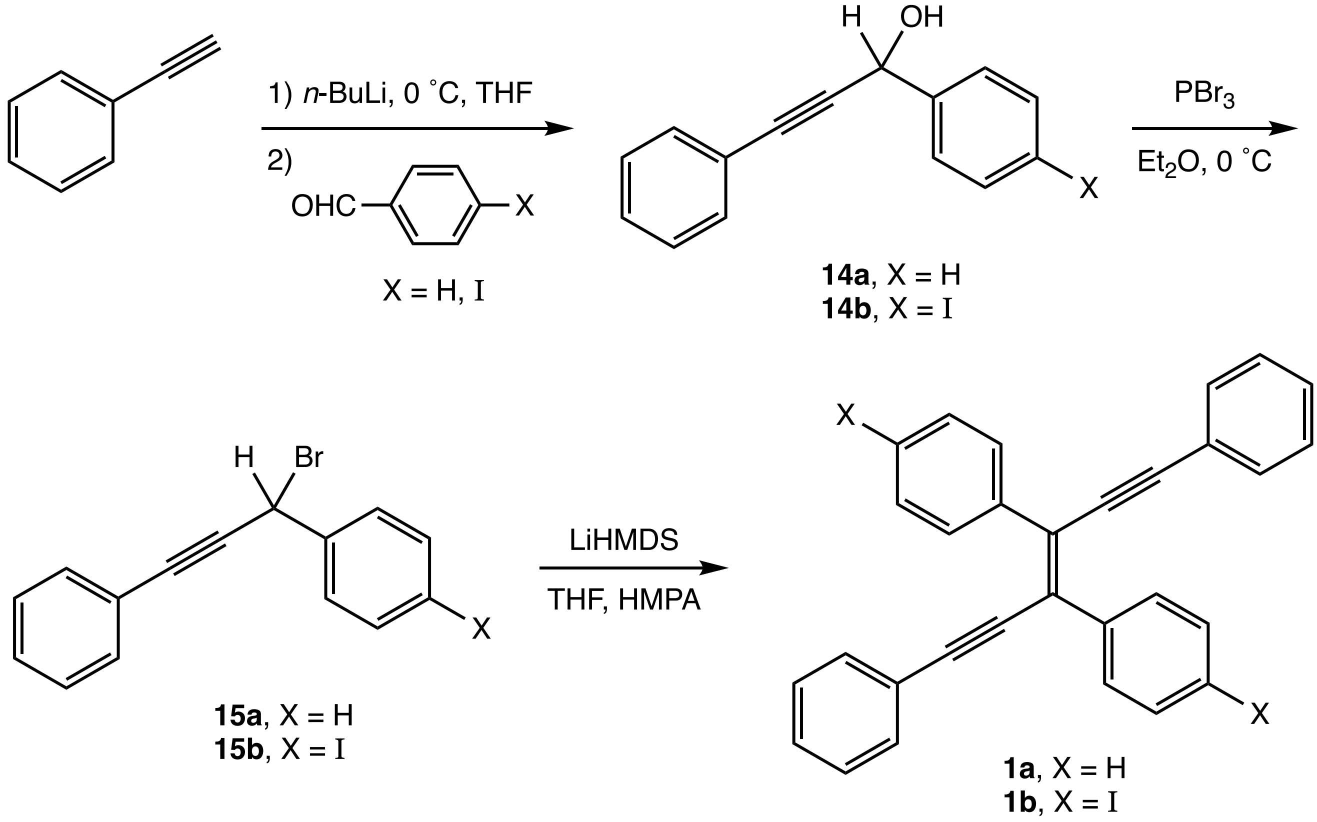

2.1.1. Experimental procedures for the preparation of (*E*)-1,3,4,6-tetraphenyl-3-hexen-1,5-diyne (1a).

a) 1,3-Diphenyl-2-propyn-1-ol (14a).[11]

Dry THF (15 mL) was added to a heat-dried 50 mL round bottom flask containing a magnetic stirring bar and placed under argon. Phenylacetylene (0.93 g, 1.0 mL, 9.1 mmol) was added via syringe. The reaction was cooled to 0 ˚C to generate the lithium phenyl acetylide. A total of 6.1 mL (9.1 mmol) of 1.5 M *n*-BuLi in THF was added dropwise via syringe. The bath was removed, allowing the reaction to warm up to room temperature. After 30 minutes, the reaction was cooled back down to –78 ˚C. Benzaldehyde (0.97 g, 0.93 mL, 9.1 mmol, 1.0 eq) was added dropwise via syringe. Once added, the bath was removed, and the reaction was left stirring at 25 ˚C for 2 hours. The reaction was quenched with saturated NH_4Cl (20 mL). The crude organic product was extracted with EtOAc, washed with brine, dried with anh. $MgSO_4$, and then evaporated on the Rotavap. The residual oil was dried under vacuum overnight. The final product

was purified as an oil via flash column chromatography (gradient from 100% hexanes to 100% CH_2Cl_2; TLC, rf = 0.50, CH_2Cl_2) to give 1.3 gram of pure 1,3-diphenylprop-2-yn-1-ol as an oil in 69% yield. ^{1}H NMR (500 MHz, $CDCl_3$) δ (ppm) 7.62 (d, J = 7.7 Hz, 2H), 7.49 – 7.46 (m, 2H), 7.41 (t, J = 7.4 Hz, 2H), 7.38 – 7.30 (m, 4H), 1.99 (bs, 1H). ^{13}C-NMR (500 MHz, $CDCl_3$) δ (ppm) 140.6, 131.7, 128.7 128.6, 128.4, 128.3, 126.7, 122.4, 88.7, 86.6, 65.1.

b) 1-Bromo-1,3-diphenyl-2-propyne (15a).[12]

A heat-dried 500 mL round bottom flask containing a magnetic stirring bar was charged with propargyl alcohol **14a** (1.0 g, 4.8 mmol) and 150 mL of dry Et_2O. The flask was cooled to 0 ˚C in an ice bath. PBr_3 (0.91 mL, 9.6 mmol, 2 eq) was added dropwise via syringe under argon and the reaction was stirred for 30 minutes. The reaction was quenched with 30 mL of sat. $NaHCO_3$. The crude organic product was extracted with Et_2O, washed with brine, and the separated organic layer dried with anhydrous $MgSO_4$, and the residual oil dried overnight at 25 ˚C under high vacuum. Compound **15a** (1.3 g, quant.) is unstable on silica gel. This crude material was carried onto the next step without purification due to its extreme sensitivity.

c) (*E*)-1,3,4,6-Tetraphenyl-3-hexen-1,5-diyne (1a).

A 250 mL round bottom flask containing 1.3 g (4.8 mmol) of crude propargylic bromide (**15a**) was charged with a magnetic stirring bar and dry THF (45 mL) and placed under argon. Separately, dry THF (20 mL) was added to another flame-dried 50 mL round bottom flask containing a magnetic stirring bar. This reaction vessel was cooled to –10 ˚C and 1.0 M LiHMDS (5.3 mL, 5.3 mmol, 1.1 eq) and HMPA (0.9 mL, 5.3 mmol, 1.1 eq) were added via syringe under argon. The reaction vessel containing the crude propargylic bromide **15a** was cooled to –90 ˚C using a frozen hexanes slurry generated using liquid nitrogen. The LiHMDS and HMPA mixture was added dropwise via syringe transfer over 30 minutes, then the reaction was left to stir and warm to room temperature. The reaction was quenched with saturated NH_4Cl (100 mL). The crude organic product was extracted with chloroform, washed with brine, and dried under vacuum. Pure iridescent white crystals of **1a** (510 mg, 56%) were isolated by slow evaporation from chloroform. It was recrystallized twice more from chloroform for the STM and nc-AFM experiments. ^{1}H NMR (500 MHz, $CDCl_3$): δ (ppm) 8.01 (d, J = 7.1 Hz, 4H), 7.49 (t, J = 7.5 Hz, 4H), 7.42 (t, J = 7.9 Hz, 2H), 7.38 (s, 2H), 7.34 – 7.28 (s, 10H). ^{13}C-NMR (500 MHz, $CDCl_3$): δ (ppm) 139.0, 131.4, 129.2, 128.6, 128.4, 128.3, 127.8, 123.3, 98.5, 90.9.

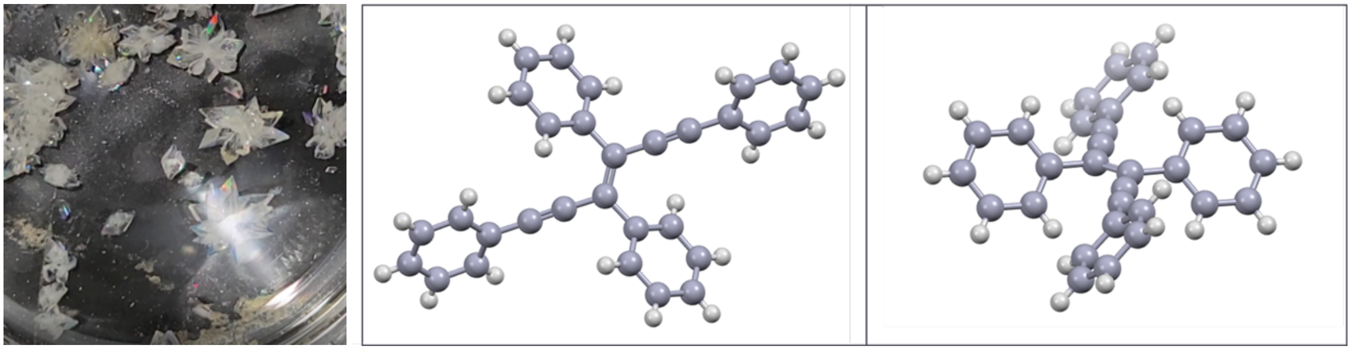

Photograph of crystals of **2a** and two representations of its X-ray crystal structure.

2.1.2. Experimental procedures for the preparation of (*E*)-3,4-bis(4-iodophenyl)-1,6-diphenyl-3-hexen-1,5-diyne (1b).

a) 1-(4-Iodophenyl)-3-phenylprop-2-yn-1-ol (14b)

Dry THF (15 mL) was added to a heat-dried 50 mL round bottom flask containing a magnetic stirring bar and placed under argon.

Phenylacetylene (0.93 g, 1.0 mL, 9.1 mmol) was added via syringe. The reaction was cooled to 0 ˚C to generate the lithium phenyl acetylide. A total of 6.7 mL (10.0 mmol, 1.1 eq) of 1.5 M *n*-BuLi was added dropwise via syringe. The bath was removed, allowing the reaction to warm up to room temperature. After 30 minutes, the reaction was cooled back down to –78 ˚C. Then, 2.1 g (9.1 mmol) of 4-iodobenzaldehyde dissolved in 20 mL of THF was added dropwise via syringe. Once added, the bath was removed, and the reaction was left stirring at RT for 2 hours. The reaction was then quenched with saturated NH_4Cl (20 mL). The crude organic product was extracted with EtOAc, washed with brine, dried with anhydrous $MgSO_4$, and the solvent was then evaporated on the Rotavap. The residual oil was dried under vacuum overnight. The product was purified by flash column chromatography (gradient from 100% hexanes to 100% CH_2Cl_2; TLC, rf = 0.45, CH_2Cl_2) to give 2.3 g of pure 1-(4-iodophenyl)-3-phenylprop-2-yn-1-ol **14b** as an oil in 76% yield. ^{1}H NMR (500 MHz, $CDCl_3$) δ (ppm) 7.74 (d, *J* = 8.5 Hz, 2H), 7.46 (d, *J* = 6.0 Hz, 2H), 7.41 – 7.30 (m, 5H), 2.3 (bs, 1H).

b) 1-(4-Iodophenyl)-3-phenylprop-2-yn-1-bromide (15b).

Dry Et_2O (150 mL) was added to a heat-dried 500 mL round bottom flask containing a magnetic stirring bar and 1.0 g (3.0 mmol) of propargylic alcohol **15a**. The flask was cooled to 0 ˚C using an ice bath. PBr_3 (0.57 mL, 6.0 mmol, 2 eq) was added dropwise via syringe under argon and the reaction was stirred for 30 minutes. The reaction was quenched with sat. $NaHCO_3$, (30 mL). The crude organic product was extracted with Et_2O, washed with brine, the separated organic layer dried with anhydrous $MgSO_4$, and the residual oil dried overnight under high vacuum. Compound **15b** is unstable on silica gel and this crude material was carried onto the next step.

c) (*E*)-3,4-Bis(4-iodophenyl)-1,6-diphenyl-3-hexen-1,5-diyne (1b).

A 250 mL round bottom flask containing 1.2 g (3.0 mmol) of crude propargylic bromide (**15b**) was charged with a magnetic stirring bar and dry THF (45 mL) and placed under argon. Separately, dry THF (20 mL) was added to another flame-dried 50 mL round bottom flask containing a magnetic stirring bar. This reaction vessel was cooled to –10 ˚C and 1.0 M LiHMDS (3.3 mL, 3.3 mmol, 1.1 eq) and HMPA (0.6 g, 0.6 mL, 3.3 mmol, 1.1 eq) were added via syringe under argon. The reaction vessel containing the crude propargylic bromide **15b** was cooled to –90 ˚C using a frozen hexanes slurry generated using liquid nitrogen. The mixture of LiHMDS and HMPA was added dropwise via syringe transfer over 30 minutes, then the reaction was left to stir and warm to room temperature. The reaction was quenched with saturated NH_4Cl (100 mL). The crude organic product was extracted with chloroform, washed with brine, and dried under vacuum. Compound **1b** (650 mg, 69%) was isolated as pure pale-yellow needles suitable for X-ray diffraction by layering hexanes over a solution in chloroform. It was recrystallized twice more for the STM and nc-AFM experiments.^{1}H NMR (500 MHz, $CDCl_3$): δ (ppm) 7.78 (d, *J* = 8.7 Hz, 2H), 7.71 (d, *J*

= 8.1 Hz, 4H), 7.41 (t, *J* = 7.4 Hz, 4H), 7.34 – 7.28 (m, 10H). ^{13}C-NMR (500 MHz, $CDCl_3$): δ (ppm) 128.4, 137.0, 131.4, 131.0, 128.9, 128.5, 127.6, 122.8, 99.48.

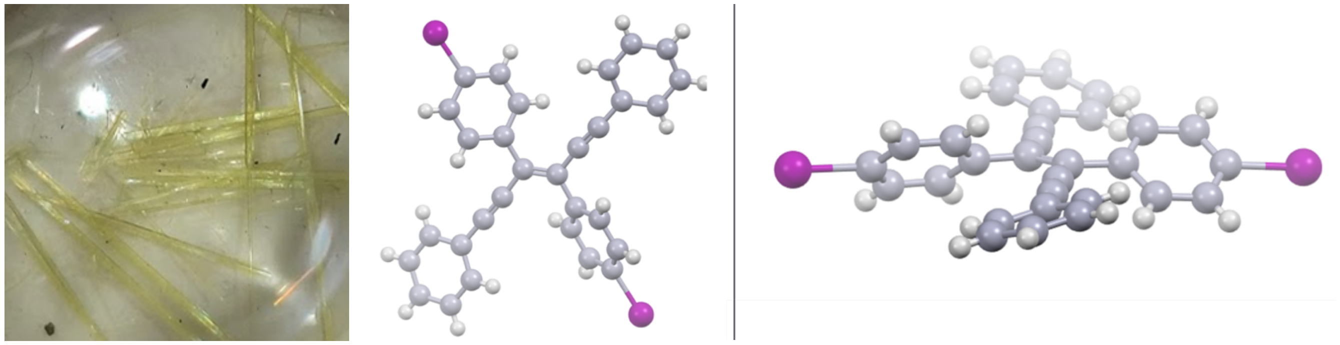

Photograph of crystals of **2b** and two representations of its X-ray crystal structure.

Section 2.2. NMR spectra.

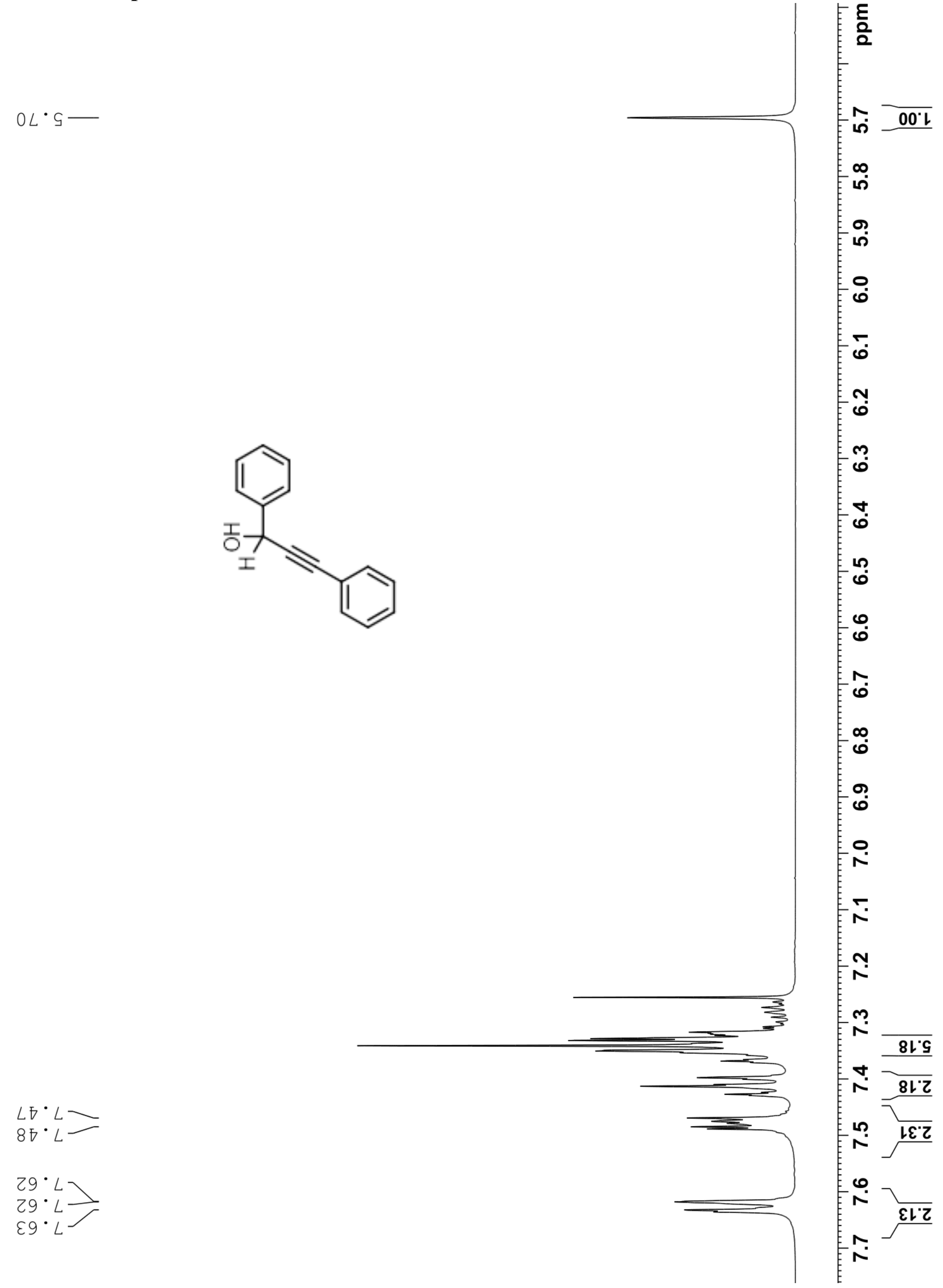

Figure S10. ^{1}H NMR of propargylic alcohol **14a** in $CDCl_3$.

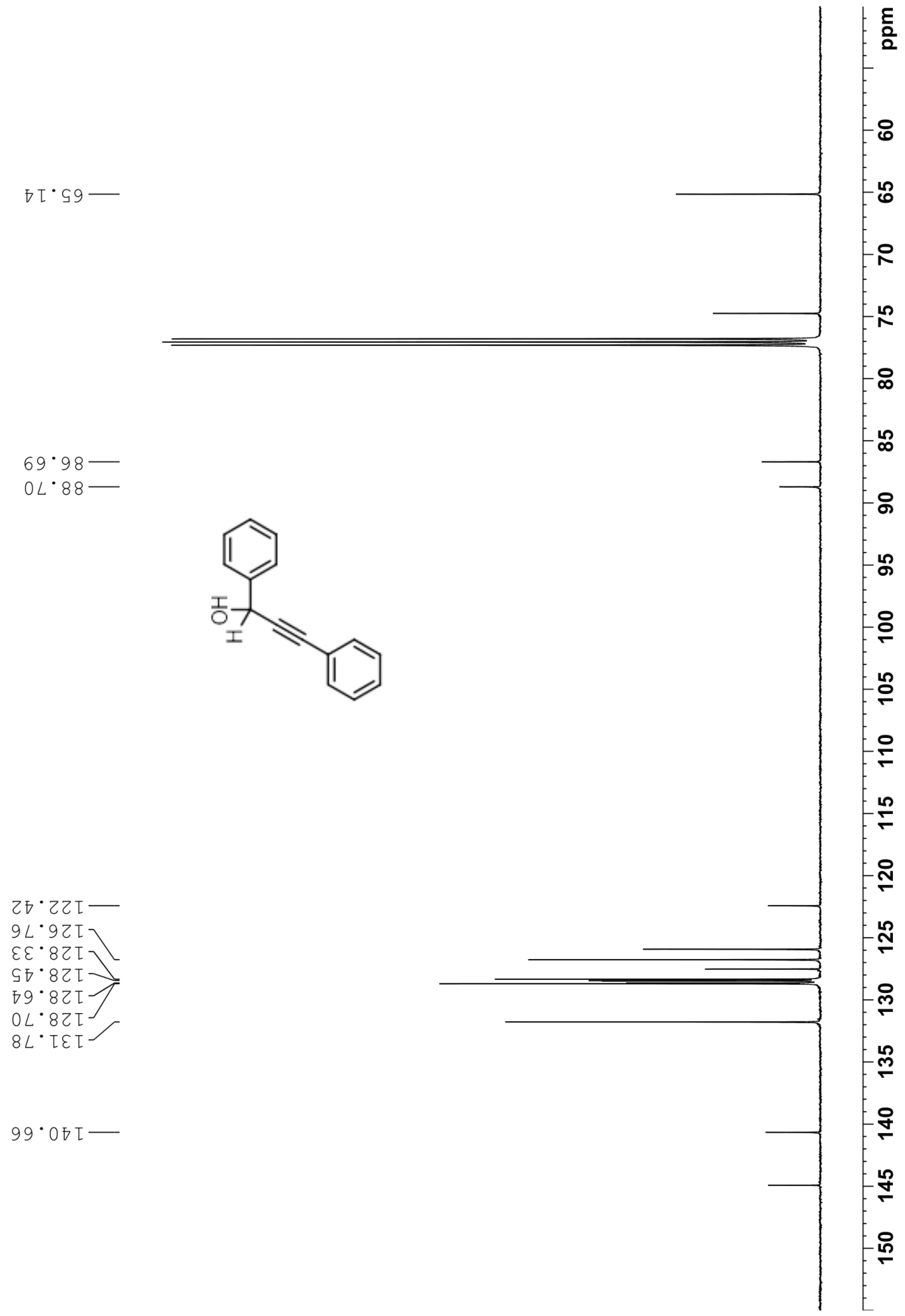

Figure S11. ^{13}C NMR of propargylic alcohol **14a** in $CDCl_3$.

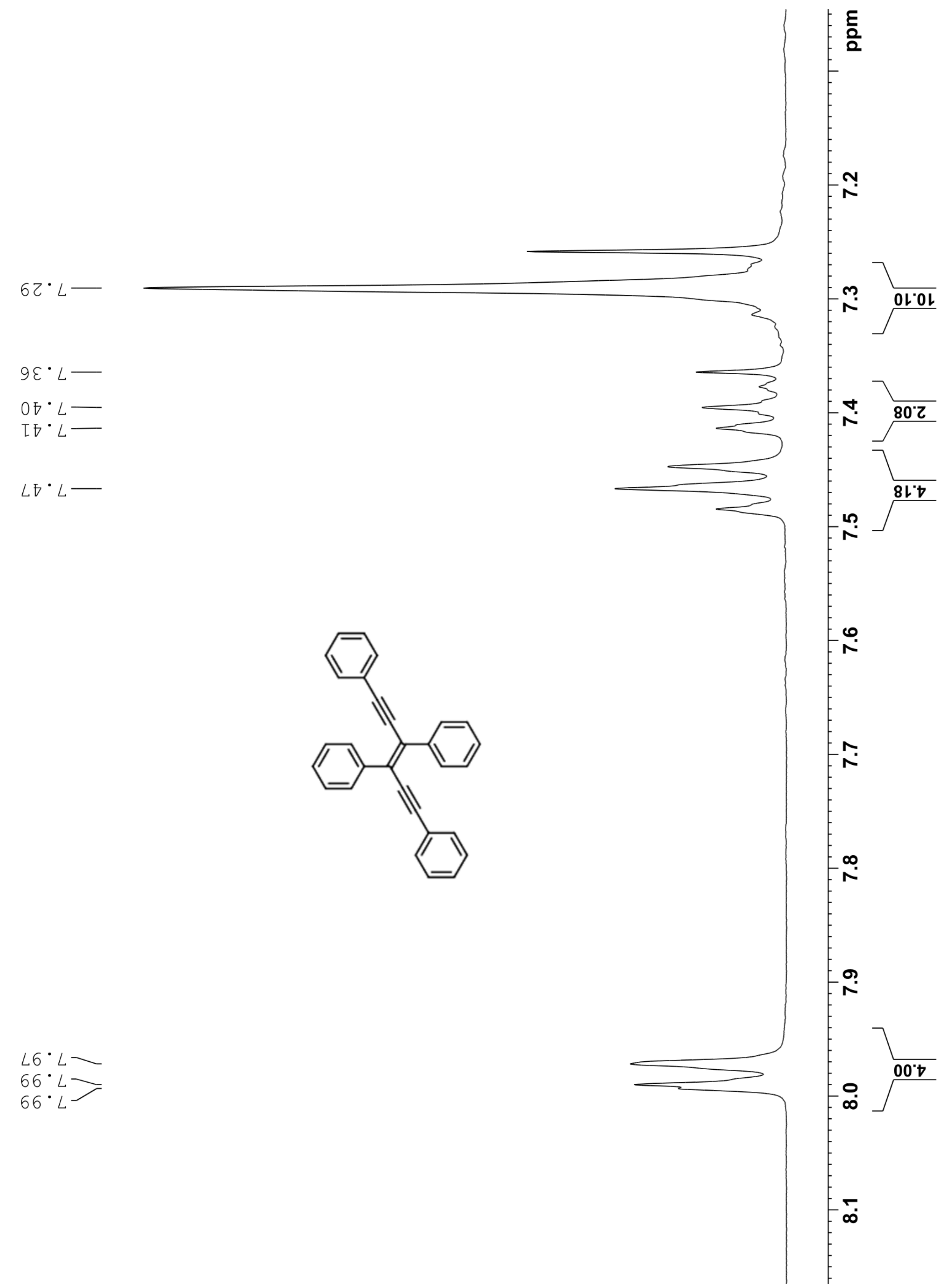

Figure S12. ^{1}H NMR of (*E*)-1,3,4,6-tetraphenyl-3-hexen-1,5-diyne (**1a**) in $CDCl_3$.

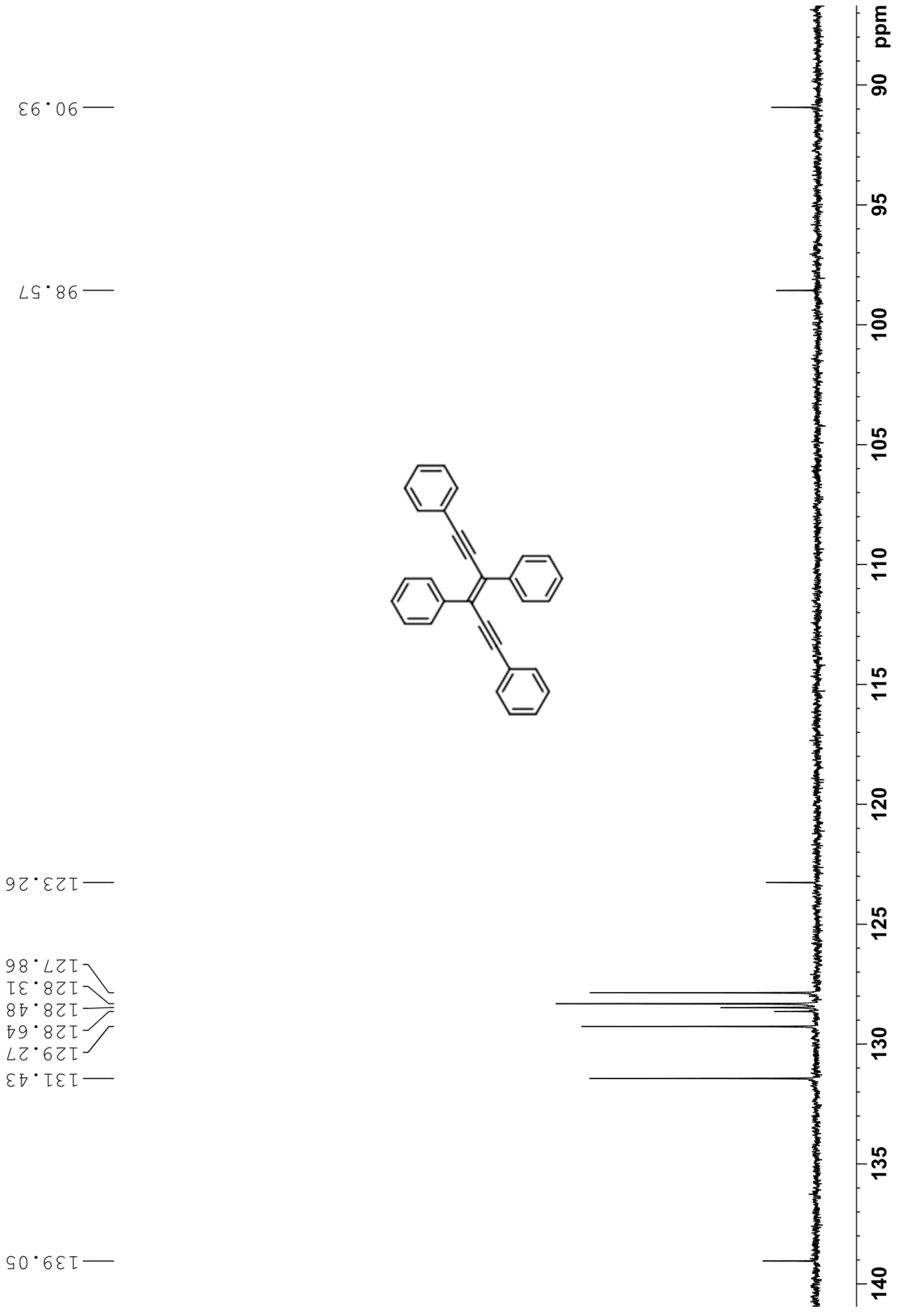

Figure S13. ^{13}C NMR of (*E*)-1,3,4,6-tetraphenyl-3-hexen-1,5-diyne (**1a**) in $CDCl_3$.

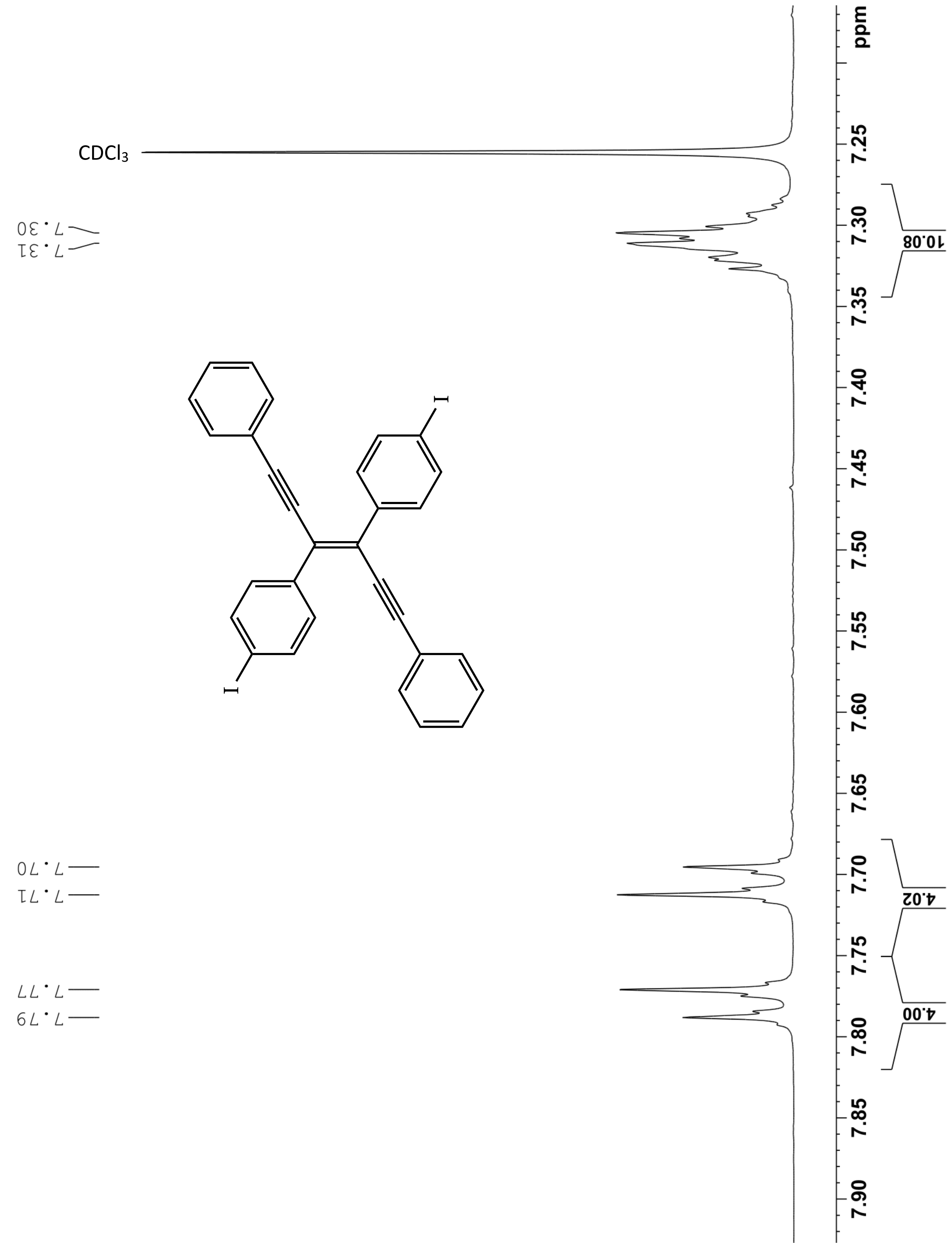

Figure S14. ^{1}H NMR of (*E*)-3,4-bis(4-iodophenyl)-1,6-diphenyl-3-hexen-1,5-diyne (**1b**) in $CDCl_3$.

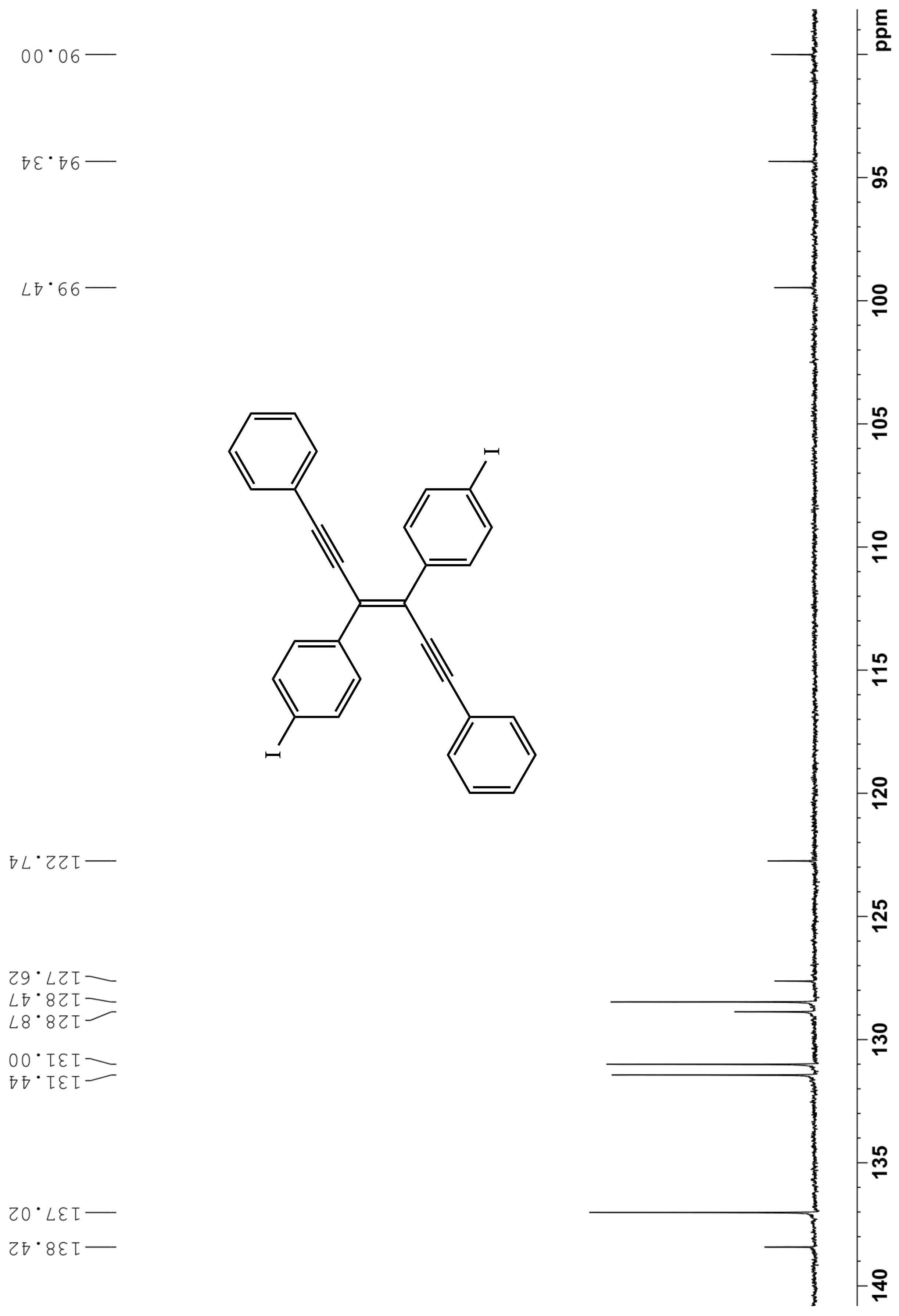

Figure S15. ^{13}C NMR of (*E*)-3,4-bis(4-iodophenyl)-1,6-diphenyl-3-hexen-1,5-diyne (**1b**) in $CDCl_3$.

2.3. STM and nc-AFM measurements

STM and nc-AFM measurements were performed in a commercial low-temperature STM from Scienta Omicron operating at a temperature of 4.5 K and a base pressure below 2×10^{-11} mbar. Au(111) single-crystal surface were prepared by Ar-ion sputtering followed by annealing at 430 ℃. Molecule **1a** was deposited on the clean Au(111) surface via molecule beam epitaxy method at 112 ℃ with a roughly rate of 0.45 Å /min. In-situ cold deposition of CO molecules was performed to get a CO-functionalized tip.

Section 3: Computational Details

3.1. Gas Phase Calculations

All unconstrained geometries were fully optimized at the B3LYP-D3/6-31G(d) level of theory in the gas phase.[13,14,15,16] All optimized geometries were verified by frequency computations as minima (no imaginary frequencies) or transition structures (one imaginary frequency). Frequency analysis was performed at 298.15 K. Subsequent single-point energy calculations on the optimized structures were performed using the M06-2X/6-311+G(d,p) level of theory.[17] All quantum chemical computations were performed with the Spartan 20 software.[18] All graphics on optimized structures were generated with CCDC's Mercury software.[5,6]

3.2. On-Surface Calculations

All DFT calculations were performed using AiiDAlab apps[19] based on the AiiDA engine[20] and the CP2K code.[21] The surface–adsorbate systems were modelled in the repeated slab scheme. The simulation cell consisted of four atomic layers of gold along the [111] direction. A layer of hydrogen atoms was used to passivate one side of the slab to suppress the Au(111) surface state. A vacuum of 40 Å was included in the simulation cell to decouple the system from its periodic replicas in the direction perpendicular to the surface. The electronic states were expanded using a TZV2P (DZVP) contracted Gaussians basis set[22] for carbon and hydrogen (gold) species. A cutoff of 600 Ry was used for the plane-wave basis set. Norm-conserving Goedecker– Teter–Hutter pseudo-potentials[23] were used to represent the frozen core electrons of the atoms. We used the Perdew–Burke–Ernzerhof parameterization for the generalized gradient approximation of the exchange-correlation functional.[24] To account for van der Waals interactions, we used the D3 scheme proposed by Grimme.[25] The gold surface was modeled using a super-cell, with a size of 38.3×32.4 Å^2 (768 gold atoms). The Au(111) slab was planar, and the herringbone reconstruction associated with this surface was not considered, as it would greatly expand the supercell and not substantially change the chemical activity of the surface.[26] To obtain the equilibrium geometries, we kept the atomic positions of the bottom two layers of the slab fixed to the ideal bulk positions, and all other atoms were relaxed until forces were lower than 0.005 eV/Å. For nc-AFM simulations using AiiDAlab, the equilibrium geometries and the electrostatic potential obtained using CP2K were used in combination with the probe particle code developed by Hapala.[27]

For the characterization of the reaction path of the cycloaromatization reaction, using advanced sampling methods such as meta-dynamics[28] would be computationally prohibitive due to the large number of atoms involved. Also relying on the nudged elastic band (NEB)[29] or string method[30] for such a long series of reaction steps would have a computational cost that we cannot afford. In order to nevertheless obtain a reasonable estimate of the reaction barriers, we use constrained geometry optimizations by defining a collective variable (length of a bond between two atoms or amplitude of a dihedral angle) for each step of the reaction pathway based on the experimentally verified structures (**1a**, **2**, and **3**). This collective variable was varied with a fine step size to sample the potential energy with adequate accuracy. The energy profile resulting from such a series of constrained geometry optimizations, where all atomic degrees of freedom were allowed to relax subject to the constraint of the collective variable, provided a reasonable estimate of the reaction barrier. Six series of constrained geometry optimizations are carried out: a) Closing the C_1-C_6 bond, b) H-migration from C_6 to C_2 after the C_1-C_6 bond formation, c) Closing the C_{14}-C_{26} bond, d) H-migration from C_{26} to C_{13} after the C_{14}-C_{26} bond formation in the main text to predict the transition states and intermediate states during the Hopf cyclization; e) Closing C_2-C_6 bond, and f) H migration after C_2-C_6 bond formation in Fig. 3. Intermediate geometries **4c** and **6b** (Fig. 3) were obtained by further optimizing without constraints the endpoints of the corresponding sequences of constrained optimizations.

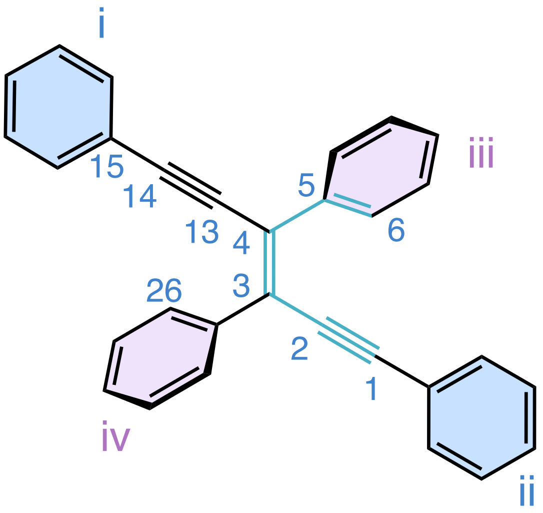

Owing to space limitation, all the coordinates for the calculated structures can be obtained from Materials Cloud platform (https://doi.org/10.24435/materialscloud:62-ew).

3.3. Band Structure Projected Density of States Calculations

The band structure of graphene nanoribbon **13** was computed by means of an AiiDAlab[19] app based on AiiDA[31] workflows and the Quantum ESPRESSO code.[32] The plane wave cutoff for the wavefunction

(charge density) was 50 Ry (400 Ry) and the PBE GGA[24] parameterization was used for the exchange-correlation functional. The ionic potential was represented with ultrasoft pseudopotentials from the Standard solid-state pseudopotentials precision library.[33] The atomic positions of the graphene nanoribbon **13**, as well as the length of the simulation cell, were fully relaxed until forces were lower than 10^–4 Rydberg/bohr, and pressure was zero. The final size of the simulation cell along the nanoribbon axis was 10.57 Å, and 15.0 Å of vacuum was added in the directions perpendicular to the ribbon axis to avoid interaction among the periodic replica. The Brillouin zone was sampled with a grid of 21 k-points.

Section 4: References

1 Pranckevicius, C.; Liu, L. L.; Bertrand, G.; Stephan, D. W. Synthesis of a Carbodicyclopropenylidene: A Carbodicarbene Based Solely on Carbon. *Angew. Chem. Int. Ed.* **2016**, *55*, 5536–5540. https://doi.org/10.1002/anie.201600765

2 Fürstner, A.; Alcarazo, M.; Goddard, R.; Lehmann, C. W. Coordination Chemistry of Ene-1,1-diamines and a Prototype "Carbodicarbene." *Angew. Chem. Int. Ed* **2008**, *47*, 3210–3214. https://doi.org/10.1002/anie.200705798

3 Pranckevicius, C.; Liu, L.; Bertrand, G.; Stephan, D. W. CCDC 1481038: Experimental Crystal Structure Determination, **2016**. DOI: 10.5517/ccdc.csd.cc1lq4fw

4 Furstner, A.; Alcarazo, M.; Goddard, R.; Lehmann, C.W. CCDC 670973: Experimental Crystal Structure Determination, **2008**. DOI: 10.5517/ccqj69q

5 Mercury 4.0: from visualization to analysis, design and prediction. C. F. Macrae, I. Sovago, S. J. Cottrell, P. T. A. Galek, P. McCabe, E. Pidcock, M. Platings, G. P. Shields, J. S. Stevens, M. Towler and P. A. Wood, *J. Appl. Cryst*. **2020** *53*, 226–235. DOI: https://doi.org/10.1107/S1600576719014092

6 https://www.ccdc.cam.ac.uk/solutions/software/

7 a) Rupf, S.; Pröhm, P.; Malischewski, M. The [2+2] Cycloaddition Product of Perhalogenated Cyclopentadienyl Cations: Structural Characterization of Salts of the $[C_{10}Cl_{10}]^{2+}$ and $[C_{10}Br_{10}]^{2+}$ Dications. *Chem. Commun.* **2020**, *56*, 9834–9837. https://doi.org/10.1039/d0cc04226a b) Duttwyler, S.; Zhang, Y.; Linden, A.; Reed, C. A.; Baldridge, K. K.; Siegel, J. S. Synthesis and Crystal Structure of a Silyl-Stabilized Allyl Cation Formed by Disruption of an Arene by a Protonation–Hydrosilylation Sequence. *Angew. Chem. Int. Ed.* **2009**, *48*, 3787–3790. https://doi.org/10.1002/anie.200900098

8 Susanne Margot Rupf, Patrick Pröhm, Moritz Malischewski CCDC 2009908: Experimental Crystal Structure Determination, 2020. DOI: 10.5517/ccdc.csd.cc25ggrw

9 Susanne Margot Rupf, Patrick Pröhm, Moritz Malischewski CCDC 2009907: Experimental Crystal Structure Determination, 2020. DOI: 10.5517/ccdc.csd.cc25ggqv

10 S. Duttwyler, Yun Zhang, A. Linden, C.A. Reed, K.K. Baldridge, J.S. Siegel CCDC 715200: Experimental Crystal Structure Determination, 2010. DOI: 10.5517/ccs06zy

11 R.S. Jordan. Setting the Record Straight: Bottom-Up Carbon Nanostructures via Solid-State Reactions. Doctoral Dissertation, University of California Los Angeles, Los Angeles, CA **2017**.

12 Jones, G. B.; Wright, J. M.; Plourde, G. W.; Hynd, G.; Huber, R. S.; Mathews, J. E. A Direct and Stereocontrolled Route to Conjugated Enediynes. *J. Am. Chem. Soc.* **2000**, *122*, 1937–1944.

https://doi.org/10.1021/ja993766b

13 Becke, A. D. Density-functional Thermochemistry. III. The Role of Exact Exchange. *J. Chem. Phys.* **1993**, *98*, 5648–5652. https://doi.org/10.1063/1.464913

[14] Lee, C.; Yang, W.; Parr, R. G. Development of the Colle-Salvetti Correlation-Energy Formula into a Functional of the Electron Density. *Phys. Rev. B* **1988**, *37*, 785–789.

https://doi.org/10.1103/physrevb.37.785

[15] Stephens, P. J.; Devlin, F. J.; Chabalowski, C. F.; Frisch, M. J. Ab Initio Calculation of Vibrational Absorption and Circular Dichroism Spectra Using Density Functional Force Fields. *J. Phys. Chem.* **1994**, *98*, 11623–11627. https://doi.org/10.1021/j100096a001

[16] Vosko, S. H.; Wilk, L.; Nusair, M. Accurate spin-dependent electron liquid correlation energies for local spin density calculations: a critical analysis. https://cdnsciencepub.com/doi/abs/10.1139/p80-159

[17] Zhao, Y.; Truhlar, D. G. Computational Characterization and Modeling of Buckyball Tweezers: Density Functional Study of Concave–Convex Ππ Interactions. *Phys. Chem. Chem. Phys.* **2008**, *10*, 2813–2818. https://doi.org/10.1039/b717744e

[18] Shao, Y.; Molnar, L.; Jung, Y.; Kussmann, J.; Ochsenfeld, C.; Brown, S. T.; Gilbert, A.; Slipchenko, L. V.; Levchenko, S. V.; O'Neill, D. P.; Jr, R. A.; Lochan, R. C.; Wang, T.; Beran, G.; Besley, N. A.; Herbert, J. M.; Lin, C.; Voorhis, T.; Chien, S.; Sodt, A.; Steele, R. P.; Rassolov, V. A.; Maslen, P. E.; Korambath, P. P.; Adamson, R. D.; Austin, B.; Baker, J.; Byrd, E. F.; Dachsel, H.; Doerksen, R. J.; Dreuw, A.; Dunietz, B. D.; Dutoi, A. D.; Furlani, T. R.; Gwaltney, S. R.; Heyden, A.; Hirata, S.; Hsu, C.-P.; Kedziora, G.; Khalliulin, R. Z.; Klunzinger, P.; Lee, A. M.; Lee, M. S.; Liang, W.; Lotan, I.; Nair, N.; Peters, B.; Proynov, E. I.; Pieniazek, P. A.; Rhee, Y.; Ritchie, J.; Rosta, E.; Sherrill, D. C.; Simmonett, A. C.; Subotnik, J. E.; III, L. H.; Zhang, W.; Bell, A. T.; Chakraborty, A. K.; Chipman, D. M.; Keil, F. J.; Warshel, A.; Hehre, W. J.; III, H. F.; Kong, J.; Krylov, A. I.; Gill, P. M.; Head-Gordon, M. Advances in Methods and Algorithms in a Modern Quantum Chemistry Program Package. *Physical Chemistry Chemical Physics* **2006**, *8*, 3172–3191. https://doi.org/10.1039/b517914a

[19] Yakutovich, A. V.; Eimre, K.; Schütt, O.; Talirz, L.; Adorf, C. S.; Andersen, C. W.; Ditler, E.; Du, D.; Passerone, D.; Smit, B.; Marzari, N.; Pizzi, G.; Pignedoli, C. A. AiiDAlab – an Ecosystem for Developing, Executing, and Sharing Scientific Workflows. *Comput. Mater. Sci.* **2021**, *188*, 110165. https://doi.org/10.1016/j.commatsci.2020.110165

[20] Pizzi, G.; Cepellotti, A.; Sabatini, R.; Marzari, N.; Kozinsky, B. AiiDA: Automated Interactive Infrastructure and Database for Computational Science. *Comput. Mater. Sci.* **2016**, *111*, 218–230. https://doi.org/10.1016/j.commatsci.2015.09.013

[21] Hutter, J.; Iannuzzi, M.; Schiffmann, F.; VandeVondele, J. Cp2k: Atomistic Simulations of Condensed Matter Systems. *Comput. Mol. Sci.* **2014**, *4*, 15–25. https://doi.org/10.1002/wcms.1159

[22] VandeVondele, J.; Hutter, J. Gaussian Basis Sets for Accurate Calculations on Molecular Systems in Gas and Condensed Phases. *J. Chem. Phys.* **2007**, *127*, 114105. https://doi.org/10.1063/1.2770708

[23] Goedecker, S.; Teter, M.; Hutter, J. Separable Dual-Space Gaussian Pseudopotentials. *Phys. Rev. B* **1995**, *54*, 1703–1710. https://doi.org/10.1103/physrevb.54.1703

[24] Perdew, J. P.; Burke, K.; Ernzerhof, M. Generalized Gradient Approximation Made Simple. *Phys. Rev. Lett.* **1996**, *77*, 3865–3868. https://doi.org/10.1103/physrevlett.77.3865

[25] Grimme, S.; Antony, J.; Ehrlich, S.; Krieg, H. A Consistent and Accurate Ab Initio Parametrization of Density Functional Dispersion Correction (DFT-D) for the 94 Elements H-Pu. *J. Chem. Phys.* **2010**, *132*, 154104. https://doi.org/10.1063/1.3382344

[26] Hanke, F.; Björk, J. Structure and Local Reactivity of the Au(111) Surface Reconstruction. *Phys. Rev. B* **2013**, *87*, 235422. https://doi.org/10.1103/physrevb.87.235422

[27] Hapala, P.; Kichin, G.; Wagner, C.; Tautz, F. S.; Temirov, R.; Jelínek, P. Mechanism of High-Resolution STM/AFM Imaging with Functionalized Tips. *Phys. Rev. B* **2014**, *90*, 085421. https://doi.org/10.1103/physrevb.90.085421

[28] Laio, A.; Parrinello, M. Escaping Free-Energy Minima. *Proc. Natl. Acad. Sci.* **2002**, *99*, 12562–12566. https://doi.org/10.1073/pnas.202427399

[29] Henkelman, G.; Uberuaga, B. P.; Jónsson, H. A Climbing Image Nudged Elastic Band Method for Finding Saddle Points and Minimum Energy Paths. *J. Chem. Phys.* **2000**, *113*, 9901–9904. https://doi.org/10.1063/1.1329672

[30] E, W.; Ren, W.; Vanden-Eijnden, E. String Method for the Study of Rare Events. *Phys. Rev. B* **2002**, *66*, 052301. https://doi.org/10.1103/physrevb.66.052301

[31] Huber, S. P.; Zoupanos, S.; Uhrin, M.; Talirz, L.; Kahle, L.; Häuselmann, R.; Gresch, D.; Müller, T.; Yakutovich, A. V.; Andersen, C. W.; Ramirez, F. F.; Adorf, C. S.; Gargiulo, F.; Kumbhar, S.; Passaro, E.; Johnston, C.; Merkys, A.; Cepellotti, A.; Mounet, N.; Marzari, N.; Kozinsky, B.; Pizzi, G. AiiDA 1.0, a Scalable Computational Infrastructure for Automated Reproducible Workflows and Data Provenance. *Sci. Data* **2020**, *7*, 300. https://doi.org/10.1038/s41597-020-00638-4

[32] Giannozzi, P.; Baseggio, O.; Bonfà, P.; Brunato, D.; Car, R.; Carnimeo, I.; Cavazzoni, C.; Gironcoli, S. de; Delugas, P.; Ruffino, F. F.; Ferretti, A.; Marzari, N.; Timrov, I.; Urru, A.; Baroni, S. Quantum ESPRESSO toward the Exascale. *J. Chem. Phys.* **2020**, *152*, 154105.

https://doi.org/10.1063/5.0005082

[33] Gianluca Prandini, Antimo Marrazzo, Ivano E. Castelli, Nicolas Mounet, Elsa Passaro, Jusong Yu, Nicola Marzari, A Standard Solid-State Pseudopotentials (SSSP) library optimized for precision and efficiency, Materials Cloud Archive 2023.65 (2023).
